# Development of a new set of Heuristics for the evaluation of Human-Robot Interaction in industrial settings: Heuristics Robots Experience (HEUROBOX)

**DOI:** 10.3389/frobt.2023.1227082

**Published:** 2023-08-31

**Authors:** Ainhoa Apraiz, Jose Antonio Mulet Alberola, Ganix Lasa, Maitane Mazmela, Hien Ngoc Nguyen

**Affiliations:** ^1^ Design Innovation Centre, Faculty of Engineering, Mechanical and Industrial Production, Mondragon Unibertsitatea, Arrasate, Spain; ^2^ Institute of Intelligent Industrial Technologies and Systems for Advanced Manufacturing (STIIMA), National Research Council (CNR), Milan, Italy; ^3^ Mechanical and Industrial Engineering Department, Università di Brescia, Brescia, Italy

**Keywords:** human-robot collaboration (HRC), human-robot interaction (HRI), user experience (UX), technology acceptance, heuristic evaluation, industry 5.0, human-centered

## Abstract

Humans and robots will increasingly have to work together in the new industrial context. Therefore, it is necessary to improve the User Experience, Technology Acceptance, and overall wellbeing to achieve a smoother and more satisfying interaction while obtaining the maximum performance possible out of it. For this reason, it is essential to analyze these interactions to enhance User Experience. The heuristic evaluation is an easy-to-use, low-cost method that can be applied at different stages of a design process in an iterative manner. Despite these advantages, there is rarely a list of heuristics in the current literature that evaluates Human-Robot interactions both from a User Experience, Technology Acceptance, and Human-Centered approach. Such an approach should integrate key aspects like safety, trust, and perceived safety, ergonomics and workload, inclusivity, and multimodality, as well as robot characteristics and functionalities. Therefore, a new set of heuristics, namely, the HEUROBOX tool, is presented in this work in the form of the HEUROBOX tool to help practitioners and researchers in the assessment of human-robot systems in industrial environments. The HEUROBOX tool clusters design guidelines and methodologies as a logic list of heuristics for human-robot interaction and comprises four categories: Safety, Ergonomics, Functionality, and Interfaces. They include 84 heuristics in the basic evaluation, while the advanced evaluation lists a total of 228 heuristics in order to adapt the tool to the evaluation of different industrial requirements. Finally, the set of new heuristics has been validated by experts using the System Usability Scale (SUS) questionnaire and the categories has been prioritized in order of their importance in the evaluation of Human-Robot Interaction through the Analytic Hierarchy Process (AHP).

## 1 Introduction

An increasing number of robots have been incorporated over the last years into Industry 5.0 (Karabegović et al., 2020; Karabegović et al., 2022; Marvel et al., 2020), which central pillar is the wellbeing of workers. As a result, operators must interact with them daily while promoting their wellbeing (European Commission, 2021). It is, therefore, important that they become partners in their everyday lives in a symbiotic manner so that they can have a natural, fluid, and satisfying experience (Kahn Jr et al., 2007; Boden et al., 2017; Chen et al., 2020; Lindblom and Alenljung, 2020). In recent years, the potential for humans and robots to work together has been recognized as a viable approach to support human workers by taking on hazardous and physically demanding tasks (Colim et al., 2021). This symbiotic relationship leverages the respective strengths of both parties to create a robust collaborative framework that enhances productivity, flexibility, and the generation of new job opportunities rather than displacing human labor (Villani et al., 2017).

Collaborative robots are particularly suited to addressing challenges related to manufacturing and assembly tasks, as they can interact physically with humans in a shared workspace (Guerin et al., 2015; Matheson et al., 2019). Conversely, human intervention remains indispensable in ensuring a high degree of adaptability and proactive responsiveness to the constantly changing demands for product customization (Prati et al., 2021). Robotic systems possess the ability to supplement and augment human sensory, physical, and cognitive attributes, while human operators can attend to the more intricate and complex cognitive tasks. Therefore, the human role will remain relevant in Human-Robot Interaction.

Prior research offers various interpretations of Human-Robot Interaction (HRI). This investigation aligns with the perspective presented by Schmidtler et al. (2015) who proposed that HRI can be categorized into three distinct types.

• *Human-robot coexistence*, denoting two entities–human and robot–operating simultaneously within a shared workspace.
• *Human-robot cooperation*, signifying both entities working together in the same environment towards a common goal.
• *Human-robot collaboration (HRC)*, which additionally requires direct physical interaction between the two entities.

Industrial and scientific research on HRI tends to be predominantly oriented towards technical issues and technological solutions (Prati et al., 2021). This “robot-centric” approach may result in limited consideration of human factors, which are critical for successfully implementing and accepting robotic systems in real-world settings. To address this gap in research, greater attention is needed on understanding the human aspects of interaction with robots, including user needs, expectations, and preferences, to ensure that robotic systems are designed in a way that is compatible with human capabilities and limitations. Therefore, it is necessary to have a human-centered approach, as it is essential to know how human’s perceptions improve productivity.

Nowadays, the interaction between human operators and robots are an unresolved concern across various application domains, ranging from healthcare and surgical procedures to personal services, with a particular emphasis on industrial contexts (Prati et al., 2021). Undoubtedly, the integration of robots into industrial and manufacturing processes holds significant advantages. Primarily, the incorporation of robots is aimed at reducing the physical and cognitive burden on human workers. This necessitates the reorganization of human activities to allocate more cognitive and supervisory responsibilities to humans while delegating repetitive and high-precision tasks that require rapidity and repeatability to robots (Probst et al., 2015).

The analysis of HRI variables is of great importance in robotics, as it allows for a comprehensive understanding of the dynamics of interactions between humans and robots. By examining factors such as communication, task allocation, and reliability between humans and robots, researchers can identify potential challenges and opportunities for improving collaboration and enhancing performance. Additionally, such analysis can inform the development of effective collaboration strategies and enable the design of robotic systems that better align with human needs and preferences. The growing prevalence of robotics in manufacturing underscores the importance of HRI variables. Understanding the factors that influence successful HRI can help to maximize the potential benefits of robotics while minimizing the risks and challenges associated with their use.

This paper focuses on the design for HRI, specifically in the context of modern factories where robots work alongside humans to support task execution. The research motivation stems from the desire to expand current research efforts, which have predominantly concentrated on the technological aspects of robotic systems, to encompass the human side of the collaboration. The need for such research is increasingly relevant as robotics technology advances and becomes more prevalent in industrial settings, where humans and robots must work together seamlessly to achieve optimal performance. By exploring collaboration from the human perspective, this research aims to identify effective ways to enhance collaboration and promote a more intuitive, fluid, and satisfying interaction between human and robotic agents. Thus, this study proposes an innovative instrument for assessing the degree to which the robot’s design, contextual features, and organizational characteristics align with the individual to facilitate efficient, effective, seamless, and satisfactory interactions.

This document is structured as follows. For a better understanding of the concepts, all the theoretical definitions are introduced in Section 2. The methodology followed in this research work is presented in Section 3, divided into five steps. Section 4 presents the final set of heuristics called HEUROBOX, while Section 5 includes the validation and prioritization results performed by the experts. Section 6 points at a critical discussion of the results, and finally, Section 7 presents the conclusions.

## 2 Research background

### 2.1 User Experience (UX) and technology acceptance in robotics

The ISO 9241-210 (2019) defined User Experience (UX) as the perceptions and responses of a person resulting from the use or expected use of a product, system, or service. This includes user emotions, beliefs, preferences, perceptions, physical and psychological responses, behaviors, and achievements that occur before, during, and after use (ISO 9241-210, 2019). In the domain of robotics, the concept of UX pertains to the nature and quality of the information exchange and its impacts on both the user and the system (Marvel et al., 2020; Prati et al. 2021) emphasized the importance of focusing on UX for successful interaction between humans and robots, especially in the industrial context. The research indicates that UX design plays a critical role in ensuring positive interaction and avoiding negative experiences that can lead to the rejection of robot features. Welfare et al. (2019) suggested that maintaining human factors, such as social interaction, autonomy, and problem-solving is crucial to minimize the negative impacts of human-robot team integration and ensure operator’s technology acceptance and positive experience. Prati et al. (2021) underscored the need for a precise study of UX from the initial design phase to guarantee acceptable and pleasant interactions, improving efficiency and effectiveness. Considering how the operator interacts with the robot and understanding its UX is difficult due to its complexity, particularly in the industrial sector. To this end, using UX-based techniques, a structured human-centered approach is needed to help practitioners solve technical issues by considering the user’s needs and capabilities (Prati et al., 2021).

The Technology Acceptance Model (TAM) is a widely used theoretical framework that aims to understand and predict user acceptance of technology (Davis, 1989; Davis, 1993). In the context of HRI, TAM can be leveraged to explore factors that affect users’ willingness to accept and interact with robotic agents. By examining the perceived usefulness and ease of use of robots, as well as attitudes towards them, TAM provides a comprehensive approach to understanding the dynamics of HRI. This model can offer valuable insights into the design and implementation of effective robotic systems that are better suited to meet the needs and expectations of human users. The field of technology acceptance has been studied through various models starting with Rogers’ diffusion theory in 1962, which proposed a five-step model from awareness to confirmation of new technology (Rogers et al., 2014; Bröhl et al., 2019). Nevertheless, robots are perceived and evaluated differently by humans compared to other technologies (Meissner et al., 2020). As a result of their distinctiveness, acceptance models from different domains may not be applicable in the field of HRI. Robots possess unique features such as autonomous movement and interaction with their surroundings, that set them apart from other technological devices. Additionally, they offer innovative forms of communication which make human beings expect more socially skilled and intelligent modes of communication than those provided by other technologies.

In this sense, Bröhl et al. (2019) conducted a study on the acceptance of HRI in an industrial context and found that job relevance was the most significant predictor of perceived usefulness, followed by subjective norm, output quality, and result in demonstrability in the acceptance model. Regarding anchor variables, perceptions of external control, self-efficacy, and robot anxiety were the most relevant. On the other hand, perceived enjoyment, perceived safety, and occupational safety were the best predictors of perceived ease of use, among the adjustment variables. Social, data protection and ethical implications were comparatively less relevant. The ergonomic design can have a positive influence on adjustment variables and improve the perceived ease of use and behavioral intention of HRI. The results indicate that the original technology acceptance model can be applied to the domain of HRI, with high correlation coefficients between perceived usefulness, perceived ease of use, behavioral intention, and user behavior (Bröhl et al., 2019).

In order to effectively design HRI environments, the evaluation phase serves as a crucial step in the design process as emphasized in the UX design workflow for HRI (Prati et al., 2021). It enables the identification of strengths, weaknesses, and areas for improvement, consequently contributing to enhance the overall UX. Therefore, it is essential to develop comprehensive and specific evaluation methods that help to improve workplace design (Maurice et al., 2017; Gualtieri, Palomba, et al., 2020b; Colim et al., 2020; Colim et al., 2021). These methods must take into account the industrial conditions, worker and cobots characteristics, as well as the level of interaction (Khalid et al., 2017). In a study on “User Experience Evaluation Methods” by Väänänen-Vainio-Mattila et al. (2008), a set of requirements for good UX evaluation was proposed. However, they noted that having a single method that meets all the requirements is impossible due to the existence of some contradictory or unrealistic requirements.

The literature has identified various types of measurements to assess HRI, including performance indicators, postural indicators, robot-related factors, or even emotion-related factors (Apraiz, Lasa, et al., 2023a). To capture these indicators, studies in the literature utilize physiological devices for objective user measurements, techniques such as RULA or RSI to assess ergonomics, questionnaires to gauge user perception, and performance indicators such as time, errors, or production rate, for objective task evaluation. However, in our previous study (Apraiz, Lasa, et al., 2023a), we concluded that there is a need for heuristic evaluations in industrial HRI environments, as they can help identify design problems in a quick and cost-effective manner. Despite the existence of contradictory or unrealistic requirements, heuristic evaluation was found to meet most of the outlined requirements (Väänänen-Vainio-Mattila et al., 2008). HRI experts can use heuristic evaluation to critically assess the design and its potential to meet users’ needs and expectations, thereby saving time and resources in the development process and ensuring the system is user-friendly and effective in achieving its intended goals.

As the field of HRI continues to evolve, there is an increasing need for new and innovative evaluation methods to assess the performance and effectiveness of these systems or the use of traditional methods in novel ways (Jiménez et al., 2012). This paper aims to contribute to this effort by compiling and regrouping a list of heuristics that can be used to evaluate HRI systems. By providing a comprehensive overview of these heuristics, this paper serves as a valuable resource for designers, engineers, and researchers in the field of HRI.

### 2.2 Heuristic evaluation

The heuristic evaluation is a widely used method based on evaluators’ criticism for the quick identification of design issues (Molich and Nielsen, 1990) because of its simplicity, low cost, and broad applicability. The iterative use of heuristics is critical to improving the performance and efficiency of HRI workspaces (Clarkson and Arkin, 2007), as well as the wellbeing of the operators. By utilizing heuristics, industrial designers and engineers can determine the best way for robots to approach and interact with human workers, allowing them to work together for the same common goal.

Nielsen and Molich (1990) introduced heuristic evaluation as an informal usability method in which multiple evaluators assess an interface design and provide feedback based on a set of heuristics developed to identify potential interface issues. As listed by Quiñones and Rusu (2017) heuristic evaluation can be applied at different stages of the design process and has various advantages, which include: i) low cost, ii) no need for extensive planning, iii) broad applicability, especially at earlier stages of the design process, and iv) ability to identify usability problems without the need for users. However, they also stated the following disadvantages: i) Evaluators must have experience and adequate knowledge to evaluate the product; ii) evaluators may not understand the tasks performed by the product, so it can be difficult to identify usability problems; and iii) usability problems are identified without directly giving an idea of how to solve it.

The traditional sets of heuristics are Nielsen`s 11 Heuristics, Norman`s 7 Principles of Usability (Norman, 2004), and the 8 main Ergonomic Criteria. From this, researchers have developed new heuristics for different contexts. In the industrial context, Mazmela (2020) and Aranburu Zabalo (2020) developed lists of heuristics specifically for industrial applications in Human Machine Interfaces (HMI).

Regarding heuristics and guidelines for HRI, Qbilat et al. (2021) proposed accessibility guidelines. In the study by Gualtieri, Rauch, et al. (2020c) prerequisites and design guidelines were classified to aid designers in creating safe, human-centered, and efficient collaborative assembly workplaces. Murphy and Tadokoro (2019) proposed 32 guidelines to proactively establish good human-robot interfaces. Frijns and Schmidbauer (2021) proposed a set of guidelines for designing the User Interface (UI) design for collaboration. Benitez Sandoval et al. (2018) proposed design principles for defining HRIs that included programming practices, characteristics of robots as products, interactive behaviors, and even user moral values. Furthermore, Bauer et al. (2009) provided heuristic rules for robots to ask for directions in HRI, resulting in improved interaction by reducing ambiguities, enhancing intuition, and enabling the robot to build an internal representation of the route.

#### 2.2.1 Requirements for a new list of heuristics

While there are several lists of heuristics available for evaluating robotics or HMIs from different perspectives to the authors’ knowledge there is a gap in the heuristic evaluation specific to industrial HRI environments from a UX and human-centered holistic approach. Unlike experiences with concrete interfaces, collaborative robotic environments are multimodal, meaning that interaction occurs through multiple channels. As such, all these channels must provide smooth and satisfying experiences. In addition, safety is of the utmost importance in these risky environments. In this context, the robot’s behavior influences the safety, comfort, and acceptance of the person in the robotic system. In our previous works (Apraiz et al., 2022), we analyzed and defined the dimensions that should be considered for the development of a new list of heuristics, specifically tailored for evaluating HRI in industrial environments, these are.

i) Safety, trust, and perceived safety. Ensuring safety is the key challenge in HRI design and implementation (Villani et al., 2018). The intrinsic goal of collaborative systems is to facilitate direct interaction between humans and robots. For a human-robot team to achieve its goal, humans must trust that the robot will protect the interests and welfare of all other individuals in the team (Hancock et al., 2011). Trust is particularly important as it directly affects the operator’s willingness to accept the information from robots, follow their suggestions, and benefit from the advantages offered by robotic systems. In relation to trust, perceived safety is an important factor to consider.
ii) Physical ergonomics. New robotic environments must be able to prevent worker discomfort as well as aggravation of diseases related to postural or physical conditions in workspaces, as musculoskeletal disorders constitute the largest category of work-related diseases in many industrial countries (Punnett and Wegman, 2004).
iii) Cognitive ergonomics and emotions. Experiential aspects impact on the acceptance of the system, i.e., usability, perceived safety and risk, emotions, and perception of appearance. Factors like mental workload, physical and mental stress, learnability, usefulness, efficiency, and intuitive use must be considered. Safety and perceived risk, although emotions are of great importance in this context, as are considered paramount for optimal satisfaction (Villani et al., 2018; Meissner et al., 2020). In terms of emotions, reliability, stimulation, and confidence should be considered (Meissner et al., 2020). Additionally, the robot’s attractiveness and visual aesthetics are crucial. These factors impact performance and individual satisfaction.
iv) Inclusivity. The issue of inclusivity arises in the domain of robotics, where not all users can access the robots due to their diverse characteristics such as visual, hearing, motor, or cognitive limitations. The neglect of these aspects during the design, implementation or interaction phase creates accessibility barriers, preventing users with disabilities from using the robotic system (Qbilat et al., 2021).
v) Multimodality. In collaborative robots, the use of multiple interfaces is common, giving rise to the need for a new list of heuristics to consider this aspect. These interfaces can enable diverse modes of communication, such as graphical, voice-based, or gesture-based communication, and they can vary depending on the type of device used (Prati et al., 2021). classified human-robot interfaces into four main categories: Visual displays (e.g., graphical user interfaces, augmented reality interfaces), gestures (e.g., facial and hand movements), speech and natural language (e.g., auditory, and text-based responses) and physical and haptic interactions. However, there is currently a lack of established guidelines for multimodal control, which requires further investigation.
vi) Type of robot and functionalities. In an industrial context, robots can develop different types of actions and tasks. As (Mazmela, 2020; Aranburu Zabalo 2020) stated in their lists of heuristics for industrial HMIs, they differentiated them according to the functionalities performed by the specific software. In this sense, it is possible to extrapolate them to the industrial robotics context. Thus, it would be appropriate for the list to be made up of different robot typologies and functionalities.

Therefore, the need has been identified to create a new list of heuristics to evaluate the HRI from a UX, Technology Acceptance, and human-centered approach, which considers safety, trust and perceived safety, physical ergonomics, cognitive ergonomics and emotions, inclusivity, multimodality and types of robots and functionalities. By developing a new set of heuristics for industrial HRI environments, early-stage suggestions for improving robots can result in cost and time savings (Weiss et al., 2010).

## 3 Methodology

**FIGURE 1:**
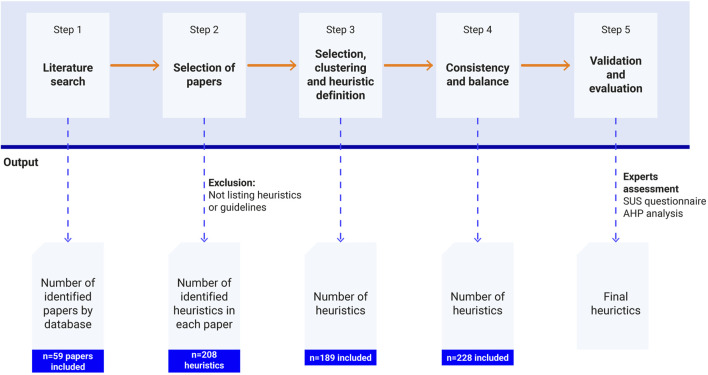
Methodology for the development of a new HRI heuristic assessment method.

This study aims to develop and validate a new list of heuristics to assess HRI in industrial contexts from a holistic approach and consider inclusiveness. The methodology employed in this study is based on the approach outlined by Quiñones and Rusu (2017), which involves identifying the application’s specific characteristics, identifying existing heuristics for its reuse -where possible-, specifying them according to the template, and finally, its validation and prioritization. When heuristics are not available and new heuristics must be developed, recommendations and design guidelines for the specific application may be of valuable interest. Figure 1 shows a summary of the methodology followed for establishing and validating the list of proposed heuristics for HRI on in industrial settings, which are further extended next.

### 3.1 Step 1: literature search

The first step aims at collecting the existing heuristics or guidelines of HRI from different databases (Table 1).

**TABLE 1:** Databases used during the literature review.

Databases	Type of database	Description
Scopus	Citation database	Scientific citation indexing service for citation searching of peer-reviewed journal articles. It is mainly used bibliometric calculations, Elsevier.
ACM Digital Library	Research database	A scientific database on subjects related to informatics and computer science.
Engineering Village	Research database	A specialized engineering database.
Science Direct	Publisher`s database	Offers its own full-text scientific journals. Covers most disciplines, but mainly focuses on science, technology and social sciences and related publishers, Elsevier.

For this purpose, a systematic literature review was carried out. The following search equation was created. Nevertheless, each database requires the adaptation of the equation according to the differences in their functionalities, as detailed in Table 2.

**TABLE 2:** Search equation and results per database.

Database	Date	Search equation	No. results
Scopus	03/06/2022	((heuristic AND evaluation) AND (((human-robot AND interaction) OR hri) OR ((human-robot AND collaboration) OR hrc)))	27
ACM Digital Library	06/06/2022	[[Abstract: “heuristic evaluation”] OR [Abstract: or] OR [Abstract: “design guidelines”] OR [Abstract: and] OR [Abstract: “human-robot interaction”] OR [Abstract: or “hri” or] OR [Abstract: “human-robot collaboration”] OR [Abstract: or “hrc”]] AND [[Title: “heuristic evaluation”] OR [Title: or] OR [Title: “design guidelines”] OR [Title: and] OR [Title: “human-robot interaction”] OR [Title: or “hri” or] OR [Title: “human-robot collaboration”] OR [Title: or “hrc”]]	2
Engineering Village	06/06/2022	(((“heuristic evaluation” OR “design guidelines”) AND (“human-robot interaction” OR “HRI” OR “human-robot collaboration” OR “HRC”)) WN AB) + (2021 OR 2020 OR 2019 OR 2018 OR 2017 OR 2016 OR 2015) WN YR	20
Science direct	06/06/2022	Title, abstract, keywords: (“heuristic evaluation” OR “DESIGN GUIDELINES”) AND (“HUMAN-ROBOT INTERACTION” OR “HRI” OR “HUMAN-ROBOT COLLABORATION” OR “HRC”)	10
		TOTAL PAPERS	59

*(“heuristic evaluation” or “design guidelines”) and (“human-robot interaction” or “hri” or “human-robot collaboration” or “hrc”)*

### 3.2 Step 2: selection of papers

After identifying previous works, a review of the literature was performed (a total of 59 works). All relevant heuristics and design guidelines in the HRI context were collected, which initially amounted to a total of 208 tentative heuristics, as seen in Table 3.

**TABLE 3:** Initial identification of heuristics and design guidelines.

Authors	Year	Title	No. heuristics or guidelines
Drury et al.	2004	Design guidelines for improved human-robot interaction	4
Clarkson and Arkin	2007	Applying heuristic evaluation to human-robot interaction systems	7
Weiss et al.	2010	A methodological adaptation for heuristic evaluation of HRI	7
Tsui et al.	2010	Developing heuristics for assistive robotics	29
Andonovski et al.	2010	Towards the development of a haptics guideline in human-robot systems	22
Keebler et al.	2012	Applying Team Heuristics to Future Human-Robot Systems	5
Adamides et al.	2014	Usability Guidelines for the Design of Robot Teleoperation: A Taxonomy	8
Manning et al.	2015	Heuristic Evaluation of Swarm Metrics’ Effectiveness	7
Coronado et al.	2017	Gesture-based robot control: Design challenges and evaluation with humans	6
Gualtieri, Monizza, et al.	2020	From Design for Assembly to Design for Collaborative Assembly - Product Design Principles for Enhancing Safety, Ergonomics and Efficiency in Human-Robot Collaboration	16
Gualtieri, Rauch, et al.	2020	Safety, Ergonomics and Efficiency in Human-Robot Collaborative Assembly: Design Guidelines and Requirements	16
Adamides	2020	Heuristic Evaluation of the User Interface for a Semi-Autonomous Agricultural Robot Sprayer	8
Qbilat et al.	2021	A Proposal of Accessibility Guidelines for Human-Robot Interaction	18
Frijns and Schmidbauer	2021	Design Guidelines for Collaborative Industrial Robot User Interfaces	24
Gualtieri et al.	2022	Development and validation of guidelines for safety in human-robot collaborative assembly systems	31
		TOTAL	208

### 3.3 Step 3: selection, clustering, and first heuristic definition

Due to the complexity of the interaction, multiple topics are covered in literature in terms of heuristic evaluation and design guidelines, i.e., functionalities, interfaces, ergonomics, and so on. For this purpose, we clustered and classified all tentative sentences collected in previous step. These groups are strategically defined by their nature and implications in the interaction to easily help and guide evaluators that might use this tool (see Table 4).

**TABLE 4:** Categories definition for the classification of design guidelines and heuristics.

Categories	Subcategories	Definition
Safety	General	Assesses generic aspects of system security, such as risk on injury or harm to users, compliance with safety standards, and the ability of the system to respond to emergencies.
	Motion planning	Assesses the system’s ability to plan and execute safe motions in the workspace, such as avoiding collisions with other objects or people, maintaining a safe distance from hazards, and optimizing movements for efficiency and safety.
	Robot systems	Analyses the characteristics of the robot system and workspaces, including the type of robot used, its capabilities and limitations, the physical layout of the workspace, and the presence of other hazards or obstacles.
	Organizational measures	Analyses the organizational measures in place to ensure safe conditions, such as training and certification programs for users, safety protocols and procedures, and the presence of safety personnel or equipment
Ergonomics	Physical ergonomics	Concerns with human anatomical, anthropometric, physiological, and biomechanical characteristics as they relate to physical activity (International Ergonomics Association, 2019) such as posture, reach, grip strength and range of motion.
	Cognitive ergonomics	Covers how well the use matches users’ cognitive capabilities, including human perception, mental processing, and memory, to ensure that the system is easy to learn, use and remember.
Functional	System	Covers the usability of the system in terms of functions available, such as the range of tasks the system can perform, the ease of accessing and using these functions, and the level of customization available to users.
	Information	Defines the assessment of how system information is presented to users, such as the clarity, relevance, and accessibility of the information, and the degree to which it supports users in performing their tasks.
	Task	Based on the efficiency of the task and how well it is implemented, such as the speed and accuracy of task performance, the level of user engagement and satisfaction, and the ability to recover from errors or interruptions.
	Error handling	Methodologies to discover, capture, and recover from errors in the system, such as error detection and correction mechanisms, user feedback and notification, and automated recovery or compensation strategies.
	Assistance	Defines documentation or help methodologies from the system, such as user manuals, online help systems, context-sensitive guidance, and in-system tutorials or demos.
Interface	General	Involves general evaluation of the interface, as well as the consistency and clarity of the interface elements and interactions.
	Visual	Analyses the interface based on information shown on displays or screens, such as the clarity and legibility of text and graphics, the use of color and contrast, and the overall visual hierarchy and organization of information.
	Gesture	Accounts for gesture inputs to the system, such as the ease and intuitiveness of gesture-based interactions, the consistency and reliability of these interactions across different users and contexts, and the ability to recover from gesture recognition errors.
	Haptic	Assess the system output through touch interactions, such as the sensitivity and precision of touch-based inputs, the feedback provided through vibration or other tactile cues, and the ability to customize or adjust haptic feedback settings.
	Voice	Evaluates the information presented and transmitted through sounds and voice, such as the clarity and intelligibility of voice prompts or instructions, the accuracy and reliability of speech recognition, and the ability to customize the voice interface setting to suit individual preferences.

In this step, all guidelines are redefined as heuristic as well. This step required a huge effort to perfectly capture the meaning of the evaluation sentence, since the complexity of interactions and multiple references might be merged in a single heuristic. For each sub-group, heuristics are clustered in two levels (basic and advanced), attending to the assessment complexity and how specific the aspect under evaluation is.

### 3.4 Step 4: consistency and balance

The present study presents a preliminary list of heuristic outcomes derived from the preceding step. Nonetheless, it is necessary to evaluate the consistency of each category and subcategory and strive towards achieving balance among them. Following this initial iteration in the heuristic development process, a targeted literature review was conducted to address any identified inconsistencies. A summary of the literature used to balance the heuristics is presented in Table 5.

**TABLE 5:** Existing specific heuristic literature to balance the new list.

Authors	Year	Title	Topic	Proposed no. heuristics
Bauer et al.	2009	Heuristic Rules for Human-Robot Interaction Based on Principles from Linguistics-Asking for Directions	Principles from Linguistic	10
Wibowo et al.	2017	Heuristic Evaluation and User Testing with ISO 9126 in Evaluating of Decision Support System for Recommendation of Outstanding Marketing Officer	Decision Support System	10
Maguire	2019	Development of a heuristic evaluation tool for voice user interface	Voice Interface	8
Andonovski et al.	2010	Towards the development of a haptics guideline in human-robot systems	Haptic Interface	11
		TOTAL		39

### 3.5 Step 5: validation and prioritization of the new set of heuristics

Once all heuristics had been defined as a draft, the list was presented to a group of expert evaluators. Evaluators were preselected according to their specific backgrounds and professional responsibilities or roles under inclusive premises. In summary, a total of 15 external evaluators were consulted according to their professional backgrounds. The invitations were sent via formal emails, which explained the topic of the research and the objective. Next, the expert survey (the pdf file in the dataset (Apraiz, Mulet Alberola, et al., 2023b)) was sent to the experts whose profiles are presented in Table 8 and the survey result was explained in Section 5 in detail.

Each recruited expert first followed a usability study through the SUS questionnaire (Brooke, 1996) that aimed at understanding the fitness of the heuristic assessment for its use in the HRI domain. This technique is well known and applied in various fields: engineering design (Gopsill et al., 2015), software engineering (Ya-feng et al., 2022) or smart PSSs (Chang et al., 2019). Besides, SUS is applicable in a small scale of survey whose questionnaire items can be easily understood by limited numbers of participants with various disciplines (Martins et al., 2015). Therefore, SUS was used to measure the perceived usability of the HEUROBOX tool under the evaluation of experts.

Moreover, the survey contained the importance rating questionnaire in the form of pairwise comparison in accordance with Analytic Hierarchy Process (AHP). This method was applied to ask the experts to grade the importance weights of each of the HEUROBOX tool’ categories: Safety, Ergonomics, Functionality, and Interfaces. These importance weights allow practitioners and researchers to prioritize which category is more important than the others, enabling them to make proper decisions in evaluating and improving human-robot systems in industrial environments. We followed the procedure of AHP provided by Saaty (2004), coded in R by Cho (2019) to evaluate the importance of each of the HEUROBOX tool’ categories in a 9-point pairwise scale, as presented in Table 6.

**TABLE 6:** Presentation of the QFD-AHP evaluation method.

A category is preferred over the left-hand category									Equally	A category is preferred over the right-hand category								
	9	8	7	6	5	4	3	2	1	2	3	4	5	6	7	8	9	
Element A																		Element B

To enhance our research transparency, we provided the expert survey (the pdf file) and the raw data of the expert responses (the xlsx file) in the dataset (Apraiz, Mulet Alberola, et al., 2023b).

## 4 Complete set of heuristics

This section contains the list of heuristics generated on the basis of the literature search and their subsequent selection and clustering. As can be seen in Table 7, HEUROBOX consists of four categories (Safety, Ergonomics, Functionality, and Interfaces). In addition, in each category, the basic level and the advanced level are differentiated. The basic level is intended to cover the essential aspects of an HRI assessment. In total, it includes 84 heuristic principles. The advanced level encompasses aspects of specific elements or functions, amounting to a total of 228 (including the ones of the basic level) heuristic principles. In the following, the development of the list by category will be explained, but it is important to note that in order to provide a smooth and satisfactory experience in a holistic manner, it is important to evaluate each category.

**TABLE 7:** The set of heuristics that compose HEUROBOX.

Category	Subcategory	Total Nº of heuristic	Nº of heuristics in the Basic evaluation	Nº of heuristics in the Advanced evaluation
Safety		**30**	**7**	**23**
	General	7	7	0
	Motion planning	10	0	10
	Robot systems	9	0	9
	Organizational measures	4	0	4
Ergonomics		**49**	**13**	**36**
	Physical ergonomics	29	4	25
	Cognitive ergonomics	20	9	11
Functionality		**78**	**36**	**42**
	System	18	6	12
	Information	28	13	15
	Task	12	7	5
	Error handling	14	7	7
	Assistance	6	3	3
Interfaces		**71**	**28**	**43**
	General	28	28	0
	Visual	22	0	22
	Voice	11	0	11
	Haptic	6	0	6
	Gesture	4	0	4
TOTAL		**228**	**84**	**144**

In table, the sum of heuristics in the category is in bold.

### 4.1 Safety

The heuristics of Safety have been fed by the heuristics and guidelines proposed by (Bauer et al., 2009; Tsui et al., 2010; Weiss et al., 2010; Gualtieri, Monizza, et al., 2020a; Gualtieri, Rauch, et al., 2020c; Gualtieri et al., 2022). The Safety category of the HEUROBOX assessment tool comprises two levels: a basic level, which includes a generic section with 7 heuristics, and an advanced level, which is divided into three subsections: Motion planning (10 heuristics), Robot systems (9 heuristics), and Organizational measures (4 heuristics). The advanced evaluation in Safety includes a total of 30 heuristics, which are intended to provide a more comprehensive assessment of the safety of HRI in assembly settings.

Based on this assessment, the system should be designed to minimize hazards related to HRI in assembly settings. These hazards include general hazards related to human-assembly parts interaction, specific mechanical hazards related to human-assembly parts interaction, and specific mechanical hazards related to robot system parts falling. The system should also be free of physical features or behaviors that could cause injury, and it should include fail-safe mechanisms to ensure safety. In addition, the system should allow for the setting of trajectories to avoid contact or entrapment of human body parts, and it should include functions to limit velocities, forces, and torques. The system should also use safety-rated soft axes and have mechanisms for energy absorption and impact force reduction, as well as sensors to anticipate or detect contact and protective features against hazards associated with the workpiece. Finally, the system should include features to prevent entrapment due to moving cables or exposed parts, and it should include signaling and highlighting to alert operators to potential hazards.

### 4.2 Ergonomics

The heuristics of Ergonomics are based on the guidelines and research by (Drury et al., 2004; Weiss et al., 2010; Adamides et al., 2014; Lewis et al., 2014; Gualtieri, Monizza, et al., 2020a; Frijns and Schmidbauer, 2021). The Ergonomic heuristics are organized into two main subcategories: i) physical ergonomics and ii) cognitive ergonomics. Each of these subcategories is further divided into two levels of assessment: i) a basic level, and ii) an advanced level. Overall, the heuristics in Ergonomics provide a comprehensive assessment of the ergonomics of HRI in assembly settings, covering both physical and cognitive demands on the operator.

• The heuristics in the physical ergonomics subcategory—4 heuristics at the basic level and 25 at the advanced level - focus on the physical demands placed on the operator during HRI, such as posture, reach, and force. These heuristics aim to ensure that the operator can perform tasks comfortably and without strain or fatigue. The advanced level of assessment in this category includes additional heuristics that address more complex issues.
• The heuristics in the cognitive ergonomics category—9 heuristics in the basic level and 11 in the advanced level - focus on the mental demands placed on the operator during HRI, such as attention, decision-making, and memory. These heuristics aim to ensure that the operator can effectively process information and make decisions while interacting with the robot. The advanced level of assessment in this category includes additional heuristics that consider more advanced cognitive demands, such as the ability to adapt to changing conditions and to learn from experience.

### 4.3 Functionality

These heuristics in the Functionality subcategory are based on the guidelines and research by (Drury et al., 2004; Clarkson and Arkin, 2007; Tsui et al., 2010; Weiss et al., 2010; Young et al., 2011; Keebler et al., 2012; Adamides et al., 2014; Lewis et al., 2014; Wibowo et al., 2017; Gualtieri, Monizza, et al., 2020a; Frijns and Schmidbauer, 2021; Qbilat et al., 2021). The Functionality heuristics are organized into five main subcategories: i) system, ii) information, iii) task, iv) error handling, and v) assistance. Each of these subcategories is further divided into two levels of assessment: i) a basic level, and ii) an advanced level.

• The System subcategory - 6 heuristics at the basic level and 13 heuristics at the advanced level - covers a wide range of issues, including the predictability and consistency of the interaction, the flexibility of the system in allowing the operator to customize and control the interaction, and the ability of the system to support the operator.
• The heuristics in the Information subcategory—13 heuristics in the basic level and 15 heuristics in the advanced level - focus on the ways in which the system provides clear, accurate and relevant information to the operator, and that it allows the operator to access the information in a convenient and efficient manner.
• The heuristics in the Task subcategory—7 heuristics in the basic level and 5 heuristics in the advanced level–aim to ensure that the system helps the operator to complete the task efficiently and effectively, without unnecessary steps or interference. These heuristics focus on simplifying various types of tasks, such as object recognition, feeding, handling and assembly, and minimizing the number of steps required to achieve goals. The heuristics also consider the ability of the system to adapt to different types of tasks and application scenarios and to allow for the reuse of previous works.
• The heuristics in the Error handling subcategory - 7 heuristics at the basic level and 7 heuristics at the advanced level–focus on the system’s ability to identify and communicate errors to the operator, as well as its ability to help the operator recover from errors. This includes using visual mechanisms to indicate errors, providing clear and understandable error messages, and offering options for recovery or reversal of actions. The heuristics also consider the system’s ability to self-inspect for damages or obstacles, and to provide information about the task environment to help the operator understand the cause of errors.
• The heuristics in the **Assistance** subcategory - 3 heuristics at the basic level and 3 heuristics at the advanced level–aim to evaluate the assistance provided by the system. It covers a range of issues related to assistance, including the ability of the system to provide clear and useful information and feedback, the use of standard rules for decision support, and the provision of context-sensitive help and documentation. Additionally, the heuristics address the importance of supporting and helping the operator to determine the most appropriate level of robotic autonomy at any given time.

### 4.4 Interfaces

The heuristics on Interfaces are based on the guidelines and research by (Drury et al., 2004; Clarkson and Arkin, 2007; Bauer et al., 2009; Andonovski et al., 2010; Weiss et al., 2010; Keebler et al., 2012; Adamides et al., 2014; Coronado et al., 2017; Maguire, 2019; Frijns and Schmidbauer, 2021; Fulfagar et al., 2021; Qbilat et al., 2021). They are organized into two levels: i) the basic level, which is composed of general issues, and ii) the advanced level, which is composed of four subcategories (visual, voice, gesture, and haptic). When evaluating a system with the advanced level, the expert evaluator must select only the subcategories that are involved in that particular system context.

• The basic evaluation level—28 heuristics - provides a general assessment of the UI in the HRI without considering the type of interface. These heuristics aim to ensure that the UI is easy and intuitive for the user to understand and use and that it helps the user to maintain appropriate awareness of the system’s state, follow task execution, and make informed decisions about their interaction with the robot.
• The heuristics for visual interfaces—22 heuristics–aim to ensure that the user interface for the HRI system is easy to use and understand and that it presents the necessary information in a clear and structured way. These heuristics cover a wide range of issues, including the use of simple graphics and icons, the minimization of multiple windows, the use of familiar language and concepts, the usability, accessibility, and aesthetics of the interface design, the appropriate presentation of sensor information, the ability to manipulate and store displayed information, the use of efficient interaction language, and the consideration of color, contrast, and visibility in the interface design.
• The heuristics for voice interface—11 heuristics - aim to ensure that the system’s voice user interface (VUI) is easy to understand and remember, efficient to use and that it provides appropriate feedback and support to the operator. These heuristics cover a range of issues, including the number of steps required in the user-system dialog, the accuracy in minimizing input errors, the natural and human-like speech, the use of efficient interaction language, and the structured dialog between the user and the system.
• The heuristics for gesture—4 heuristics–aim to ensure that gestures are intuitive and natural for the user, and that can be used to control the robot’s movements or actions.
• The haptic interface heuristics—6 heuristics–aim to ensure that the system is able to provide sufficient tactile feedback to the user through the use of haptic or tactile objects, and that the user is able to perceive and interpret this feedback correctly. These heuristics focus on the interpretability and perception of basic directions, as well as the tactile attributes of the objects used, such as texture, force, vibration, duration, and acceleration.

## 5 Experts’ validation and prioritization results

This section focuses on presenting the validation and prioritization results performed by the experts, following the methodology defined in the previous section. Nguyen Ngoc et al. (2022) highlighted a crucial gap in the existing literature regarding design tools: the need for empirical usability evaluations. This prompted us to address this limitation by conducting an assessment of the HEUROBOX tool. To achieve this goal, we followed the indications by Nguyen Ngoc et al. (2022) to perform an assessment that involved soliciting expert opinions using the System Usability Scale (SUS) and Analytic Hierarchy Process (AHP) methodologies. The presented dataset is stored at Mendeley data (Apraiz, Mulet Alberola, et al., 2023b).

As previously exposed, a total of 15 experts participated in the evaluation of HEUROBOX (see Table 8), including six female participants (40%), one non-binary individual (6,7%), and one person who chose not to disclose their gender (6,7%). Of the participants, two were practitioners (13,3%), while the remaining 86,7% were academics.

**TABLE 8:** A list of experts asked for the validation of the heuristics.

Expert number	Gender	Expertise	Major fields	Working years
Expert #1	Male	Academist	Biomedical Engineering, Biomechanics, HRI, and Reinforcement learning	3
Expert #2	Male	Academist	Computer science, Artificial Intelligence, Machine learning, and IoT	14
Expert #3	Male	Academist	Industrial Engineering, Design for manufacturing, Design for Assembly	4
Expert #4	Female	Academist	Psychology, User Experience in Virtual Reality	5
Expert #5	Female	Academist	Biomedical Engineering, Digital technologies, and user-centered design	9
Expert #6	Male	Practitioner	Robotic systems, PLC, and knowledge	1
Expert #7	Non-binary	Academist	Electronical Engineering, Control, and automation	3
Expert #8	I prefer not to say	Academist	Electronical Engineering, Control	11
Expert #9	Male	Practitioner	Robotic systems, automation, Machine tool	15
Expert #10	Female	Academist	User Experience, Technology Acceptance, HRI	4
Expert #11	Male	Academist	Mechatronics, Aerospace, Physics, Circular Economy	4
Expert #12	Female	Academist	Inclusive Design, Digital divide,	3
Expert #13	Female	Academist	User Experience, Technology Acceptance, Human Machine Interface	12
Expert #14	Male	Academist	User Experience, Virtual Reality	4
Expert #15	Female	Academist	Human-Centered Design	3

### 5.1 Usability assessment

As indicated by Nguyen et al. (2022), to measure the usability of HEUROBOX, we used a simplified version of the SUS (Brooke, 1996), a 10-item questionnaire measuring the usability perception applied on 5-point Likert response options (strongly disagree to agree strongly).

The raw data for the SUS responses can be found on the second page titled “SUS” within the dataset’s spreadsheet (the xlsx file) in the dataset (Apraiz, Mulet Alberola, et al., 2023b). The first column consists of the questionnaire items. Subsequently, the following 15 columns present the raw responses provided by the experts, with their corresponding identifications matching those in Table 8.

**FIGURE 2:**
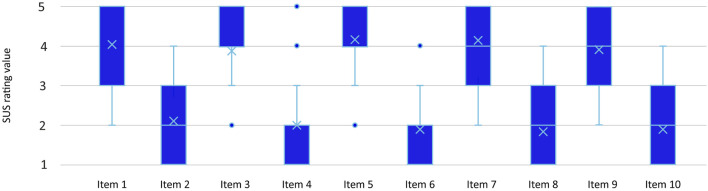
The scores obtained using the SUS questionnaire for HEUROBOX.

The SUS comprises 10 questions, with odd-numbered questions having positive meanings and even-numbered questions having negative meanings. The positive questions are scored by reducing the user’s score by one point, while the negative questions are scored by subtracting the user’s score from 2. The total scores are then multiplied by 2.5 to obtain a range of 0–100. The mean average SUS score for HEUROBOX is 76 from experts’ perspectives. As indicated on the adjective range of SUS scores by Bangor et al. (2009), the ratings of HEUROBOX fall into “excellence”. The results in Figure 2 represent the average ratings given by the experts for each item of the SUS questionnaire.

Upon detailed examination of the data presented in Figure 2, it is evident that the odd-ordered items in the System Usability Scale (SUS) obtained higher average rating values above 3, indicating an upbeat assessment of the usability of HEUROBOX.

Overall, the evaluation of HEUROBOX by experts was positive, as evidenced by the high average rating of 4.05 for the item “I would use HEUROBOX”. The evaluators also gave an average rating of 4 for the item “HEUROBOX was easy to use” and 3.9 for the item “I felt confident using HEUROBOX,” indicating ease of use and confidence in using the system.

However, the evaluation also highlighted areas of concern among experts’ regarding the system’s complexity. For instance, the item “HEUROBOX was too complex for me” obtained an average rating of 2.1, indicating that some participants found the system challenging. The item “HEUROBOX was hard to use” also received an average rating of 1.85, further supporting that some participants struggled to use the system. Additionally, some participants indicated the need for assistance using the system, with an average rating of 1.95 for the item “I really need help from someone to use HEUROBOX.”

Overall, the evaluation results suggest that HEUROBOX is a useable and intuitive system with scope for improvements in complexity and user support. The findings from the positively worded SUS items indicate a generally positive perception of the tool among the expert evaluators, with items related to ease of use and integration of various parts of the tool receiving higher scores than those related to complexity and user support. These findings can serve as valuable inputs to inform the design and development of HEUROBOX, to enhance its usability and user satisfaction.

### 5.2 AHP assessment

The raw data for the category-rating questionnaire can be found on the third page labeled “AHP” within the dataset’s spreadsheet (the xlsx file in the dataset (Apraiz, Mulet Alberola, et al., 2023b)). The first column of the file sheet contains the heuristic categories being rated and indicates the pairwise comparisons being evaluated. Subsequently, the succeeding 15 columns present the raw responses provided by experts using a nine-point rating scale for the pairwise comparisons. The 17th column contains the raw data in the form of CSV value strings, which are utilized as inputs for data processing in R. Finally, the last column offers a comprehensive overview of the data points, indicating that out of a total of 390 data points, there are no instances of missing data.

The R codes (Cho, 2019) for executing the AHP algorithms (Saaty, 2004) of the raw data can be found in the. html file of the dataset (Apraiz, Mulet Alberola, et al., 2023b). These R codes are presented in the four main sequenced sections: i) R package preparation, ii) data inputs, iii) calculation of aggregated importance weights and iv) calculation of the consistency ratios.

Table 9 presents the outcomes of the AHP method (Saaty, 2004) applied to experts’ responses and computed in the R programming language (Cho, 2019). The analysis reveals that Safety is the most essential category for designing HRI systems in industrial settings. It received the highest weight at 0.469, followed by Functionality (0.207), Interfaces (0.173), and Ergonomics (0.151). This suggests that, for the experts who participated in the study, it is crucial that HRI systems are designed and built in such a way as to minimize the risks of injury or damage to users, the robots themselves, and other elements of the environment.

**TABLE 9:** Aggregated importance weights on each group of HEUROBOX in accordance with AHP.

Categories	Priority	Subcategories	Priority
Safety	0.469	Motion Planning	0.353
		Robot systems	0.355
		Organizational Measures	0.291
Ergonomics	0.151	Physical ergonomics	0.464
		Cognitive ergonomics	0.536
Functionality	0.207	System	0.215
		Information	0.152
		Task	0.225
		Error handling	0.268
		Assistance	0.140
Interfaces	0.173	Visual	0.407
		Voice	0.225
		Haptics	0.173
		Gesture	0.195

Breaking down the Safety category further, the Robot Systems subcategory received the highest weight at 0.355, followed by Motion Planning (0.353) and Organizational Measures (0.291). This suggests that in order to optimize safety, design considerations for robot systems should prioritize these areas. This result suggests that designing safe robotic systems requires a focus on the robot itself, then on its movement planning and the organization of the system. Then, Motion Planning highlights the significance of designing efficient and accurate movement algorithms for robots. Proper motion planning is crucial for avoiding collisions, navigating complex environments, and ensuring smooth and precise interactions with humans and surrounding objects. Lastly, the category of Organizational Measures is ranked in third place. Although experts have assigned it relatively less importance compared to the other subcategories, its significance should not be underestimated as it encompasses various aspects related to the organization and management of the workplace. While the focus is often placed on the technical aspects of robotics, neglecting the organizational aspects can have detrimental effects on the overall performance and safety of the system.

Regarding Ergonomics, may have been ranked fourth in importance because, although it is essential to consider the HRI from an ergonomic perspective, the other categories are more critical for the overall performance and success of the robot system. It is important to note that even though ergonomics may be ranked lower in importance, it should still be given adequate attention in the design and development of robotic systems. Within the Ergonomics category, Cognitive Ergonomics was deemed more important at 0.536 than Physical Ergonomics at 0.464. This highlights the importance of designing systems that are optimized for mental workload and ease of use. It suggests that designing robotic systems that consider the cognitive aspects of HRI, such as attention, perception, and decision-making, is more critical than designing systems that focus solely on physical aspects, such as posture and movement.

In the Functionality category, the Error Handling subcategory received the highest weight at 0.268, followed by Task (0.225), System (0.215), Information (0.152), and Assistance (0.140). This indicates the importance of developing robust error-handling mechanisms and defining relevant tasks for the system. The high weight assigned to the Task subcategory indicates that experts prioritize the system’s capability to carry out specific tasks accurately and successfully, highlighting the importance of designing robotic systems that are optimized for the specific tasks they are intended to perform in industrial settings. The System subcategory’s weight suggests that experts recognize the importance of designing a well-structured and integrated system. The inclusion of Information and Assistance subcategories underscores the value of providing relevant and helpful information to users and helping when needed. Nevertheless, designing systems that can effectively provide users with the necessary information and support contributes to a more intuitive and user-friendly experience, enhancing the overall functionality of the robotic system.

The Interfaces category was placed third through experts’ opinions. The Interfaces category is considered important but not as critical as the Safety and Functionality categories. The experts may have prioritized the other categories due to their direct impact on the overall success and effectiveness of the robotic system. The Visual subcategory was deemed the most critical at 0.407, followed by Voice at 0.225, Gesture at 0.195, and Haptics at 0.173. This highlights the importance of developing interfaces that are visually clear and easy to understand. Visual cues and feedback play a crucial role in facilitating effective communication and interaction between humans and robots. By designing visually appealing and informative interfaces, users can easily understand and interpret the system’s status, actions, and intentions.

Overall, the results of the AHP analysis provide valuable insights into the relative importance of different categories and subcategories for designing robotic systems that optimize safety, functionality, interfaces, and ergonomics. Design considerations should prioritize the areas identified in the analysis to ensure a successful and user-friendly system.

The present study yielded a Consistency Ratio (CR) for each of the evaluated categories. Specifically, the general category exhibited a CR of 0.104, while the Safety category displayed a CR of 0.197. In the Ergonomics category, only one pair-wise comparison was conducted, thus rendering a NaN value for the CR. Meanwhile, the Functionality category demonstrated a CR of 0.129, and the Interfaces category exhibited the lowest CR of 0.104. Although (Saaty, 2004) allows a limit of not more than 0.1, (Ho et al. 2005), respond CR could be relaxed to 0.2 for groups with different expertise.

## 6 Discussion

In current literature, to the authors’ knowledge, there is a gap in the heuristic evaluation of HRI in industrial environments from a UX and human-centered design perspective. Thus, a holistic and human-centered approach is lacking, as the literature has been largely “robot-centric” up to this point (Prati et al., 2021). To achieve this goal, the study proposes a new instrument based on heuristic evaluation called HEUROBOX to evaluate the HRI systems, and thus, improve the UX, technology acceptance, and overall wellbeing. The proposed tool, HEUROBOX, addresses this gap by compiling heuristics proposed by various expert authors in different domains. As a result, it provides a comprehensive tool that incorporates multiple perspectives.

Therefore, HEUROBOX is a novel heuristic evaluation tool proposed for assessing the degree of alignment between the robot and contextual features with human needs to facilitate efficient and satisfactory interactions in industrial settings. The tool comprises four categories: Safety, Ergonomics, Functionality, and Interfaces. The basic level of HEUROBOX encompasses fundamental aspects of HRI evaluation and incorporates 84 heuristic principles. On the other hand, the advanced level comprises a range of specific elements or functions and includes 228 heuristic principles, including those at the basic level. It is important to note that evaluating each category is crucial for holistically ensuring a seamless and satisfactory experience.

### 6.1 The alignment of HEUROBOX with the requirements

HEUROBOX, as a comprehensive heuristic evaluation tool, has been specifically designed to fulfill the requirements outlined in Section 2.2.1 on the “Requirements for a new list of heuristics” for assessing HRI on industrial environments. Therefore, Table 10 summarizes how HEUROBOX matches with the requirements established.

**TABLE 10:** The justification of how HEUROBOX matches the requisites established in Apraiz et al. (2022).

Requirements	How HEUROBOX matches
Safety, trust, and perceived safety	HEUROBOX is designed with safety as a top priority. It incorporates a specific category of Safety (with a total of 30 heuristics) and advanced safety features, such as collision detection and avoidance, to ensure the safety of all individuals involved in the HRI. Additionally, HEUROBOX is designed to establish trust between human operators and robots, ensuring that the robot will protect the interests and welfare of all individuals on the team.
Physical ergonomics	HEUROBOX has been designed to address physical ergonomics in the workspace, ensuring that workers are comfortable and not at risk for musculoskeletal disorders. In fact, it contains a specific subcategory on physical ergonomics consisting of a total of 4 heuristics in the basic evaluation and 25 in the advanced evaluation. The system can help prevent worker discomfort and aggravation of postural or physical conditions in the workspace.
Cognitive ergonomics and emotions	HEUROBOX is designed with cognitive ergonomics and emotions in mind. In fact, it has a specific subcategory on cognitive ergonomics containing a total of 9 heuristics in the basic evaluation and 20 in the advanced evaluation. The different categories among HEUROBOX would help to improve aspects such as mental workload, physical and mental stress, learnability, usefulness, efficiency, intuitive use, reliability, stimulation, and confidence.
Inclusivity	HEUROBOX prioritizes inclusivity, recognizing that not all users can access the robots due to diverse characteristics such as visual, hearing, motor, or cognitive limitations. The system is designed with accessibility in mind, preventing the creation of barriers that would prevent users with disabilities from using the robotic system. HEUROBOX implements the accessibility guidelines by Qbilat et al. (2021) through the different categories.
Multimodality	HEUROBOX offers a variety of interfaces to enable multimodal communication, such as graphical, voice-based, or gesture-based communication. It includes visual displays, gestures, speech and natural language, and physical and haptic interactions. This range of interfaces allows users to interact with the system in the way that best suits their needs.
Type of robot and functionalities	HEUROBOX is designed to accommodate a variety of robot types and functionalities, allowing for flexibility in the workplace. It is designed to support different types of software, allowing it to perform different functionalities as needed.

### 6.2 Assessment of HEUROBOX

Usability evaluation through expert assessments of the HEUROBOX tool has obtained favorable results, indicating the effectiveness of the tool in assessing the quality of HRI in industrial settings. The positive outcomes from the usability evaluation of HEUROBOX indicate that it is a promising approach for ensuring that HRIs are efficient and satisfactory. Moreover, the general index of 76 obtained from the usability evaluation highlights the potential for HEUROBOX to provide a comprehensive evaluation of the quality of HRI, considering various factors such as safety, ergonomics, functionality, and interfaces. These findings support the use of HEUROBOX as a valuable tool for evaluating and improving HRIs in industrial settings from a human-centered perspective.

The findings discussed above provide valuable insights for designers and developers in prioritizing their design efforts to optimize critical aspects of HRI. Designers can leverage these results as a guide to focus on key areas that have been identified as important by experts in the field. For instance, the experts’ responses, obtained through applying the AHP, indicate that Safety is the most critical general category, followed by Functionality, Interfaces, and Ergonomics. The focus on safety is especially important in robotic systems that interact with human beings, such as collaborative robots, as these systems are required to be safe and reliable for working alongside people. Nevertheless, safety is one of the most studied aspects of industrial robotics, and there are numerous standards that robots must comply with to ensure safety aspects (ISO 10218, 2011).

The high weight assigned to the Robot Systems subcategory within the Safety category emphasizes the significance of considering the robot itself when designing safe robotic systems. This insight suggests that designers should pay careful attention to the robot’s construction, functionality, and inherent safety features to minimize risks and ensure user wellbeing. Similarly, the prioritization of error-handling highlights the need for robust mechanisms to handle errors or failures that may occur during system operation. By implementing effective error-handling strategies, such as error detection, recovery, and fault tolerance, the reliability and performance of the robotic system can be enhanced. As well as the emphasis on the Visual subcategory implies that designers should prioritize creating visually appealing and intuitive interfaces. This insight emphasizes the significance of clear and informative visual cues in facilitating effective communication and interaction between humans and robots.

By utilizing these insights, designers, and developers can allocate their resources and efforts strategically, focusing on the critical aspects identified by experts. This approach ensures that the HRI is optimized for safety, functionality, ergonomics, and interfaces, leading to improved UX and higher user acceptance and overall wellbeing. Overall, the results discussed in this study provide practical guidance for designers and developers, enabling them to prioritize design efforts and optimize their systems for critical aspects of HRI.

### 6.3 Strengths and limitations of HEUROBOX

In the field of HRI in industrial settings, the evaluation of UX and technology acceptance of the systems is crucial. In this sense, HEUROBOX provides a structured approach to evaluating HRI, allowing researchers, developers, or practitioners to systematically identify and assess the effectiveness of different aspects of the interaction in a quick and cheap way.

HEUROBOX provides a framework for evaluating robotic systems that are both comprehensive and systematic. Using this set of heuristics allows evaluators to identify a wide range of potential usability issues that may otherwise be missed. Additionally, the systematic nature of HEUROBOX ensures that all aspects of the HRI are considered, including both the technological and human factors involved.

Heuristics can provide valuable insights into the UX of collaboration, helping researchers and designers understand how people perceive and interact with robots. Like other heuristic evaluation tools, HEUROBOX can be used iteratively throughout the design process (Nielsen and Molich, 1990; Rubin and Chisnell, 2008). Furthermore, HEUROBOX can be used in conjunction with other evaluation methods to achieve a more comprehensive evaluation. For instance, usability tests can complement the heuristic-based evaluation by incorporating performance indicators like task completion time, error rates and production rates. Additionally, questionnaires can be utilized to gauge users’ perceptions, and physiological responses of users can be captured using devices such as electroencephalograms (EEG), electromyograms (EMG) or electrocardiograms (ECG). Combining different evaluation methods can help to provide a more well-rounded perspective and identify potential issues that may have been missed by using heuristics alone. The proposed list of heuristics is also scalable, allowing the addition of new heuristics that reflect and assess new functionalities and designs of robotic systems that affect the interactions, as well as novel aspects of the phenomena behind the HRI that must be evaluated. It can be used in conjunction with other evaluation methods for a more comprehensive evaluation. Combining different evaluation methods can help to provide a more well-rounded perspective and identify potential issues that may have been missed by using heuristics alone (Apraiz, Lasa, et al., 2023a).

While HEUROBOX can provide a precise evaluation of HRI, it also has the limitation that the evaluator must have a good understanding of the specific robot being studied. This is because HEUROBOX includes a set of detailed heuristics that require precise knowledge of the features, functions, and capabilities of the robot in question. However, this limitation can also be seen as a strength, as it allows researchers, developers, or practitioners to carry out a very specific and targeted evaluation of the HRI. Ideally, the evaluators involved in the heuristic evaluation should have a multidisciplinary background, encompassing expertise in HRI and UX. The diverse expertise ensures a comprehensive evaluation, considering both technical aspects of the system and the human factors. Heuristic evaluations sometimes could be subjective and rely on the expertise and experience of the evaluator, which can introduce bias and variability into results. Also, it can be perceived as time-consuming and labor-intensive for the evaluators. These considerations highlight the need for careful planning, execution, and interpretation of heuristic evaluations in HRI to obtain valid and meaningful results.

It is widely recognized that UX is influenced by a multitude complex factor, and individual factors play a crucial role in evaluating experiences (Prati et al., 2022). Despite the HEUROBOX tool’s primary focus on the technical aspects of the robot itself, it does consider elements that undeniably enhance the UX by taking into account factors such as physical and cognitive ergonomics, personalization, ensuring complete safety, and functional modes of the robot. By addressing these aspects, the tool aims to improve the overall experience for individuals interacting with the robot. While it may initially appear that the human aspect is somewhat overlooked, the evaluation facilitated by HEUROBOX enables the design of the robotic system in the best possible way to provide a positive experience for any individual. However, it is important to acknowledge that complementing the evaluation with other techniques, such as usability testing that specifically considers individual characteristics and preferences, is always beneficial (Apraiz, Lasa, et al., 2023a). These additional methods can further enrich the assessment of the UX and ensure that the robot’s design aligns with the diverse needs and expectations of users. By combining the insights gained from HEUROBOX with complementary techniques, a more holistic understanding of the HRI and its impact on the UX can be achieved.

Regarding multimodal interactions, which involve the integration of multiple sensory modalities for more natural communication with robots, have the potential to create powerful user interfaces and channels of interaction. While the proposed list of heuristics is applicable to different types of interfaces and, therefore, to multimodal environments, there may be overlap among the heuristics proposed within the subcategories of the Interfaces section. This overlap could lead to a situation where a robot agent with two types of interfaces may appear to fulfill a lower percentage of heuristics. However, when these two interfaces complement each other effectively, the overall level of interaction may improve accordingly. Therefore, it is important to consider that the evaluation of interfaces in a multimodal context requires a nuanced approach. The overlap among heuristics should be carefully examined, taking into account the interplay between different modalities and how they contribute to the overall UX.

Despite these limitations, HEUROBOX has the potential to provide a precise evaluation of HRI. Its detailed set of heuristics requires evaluators to have a good understanding of the specific robot being studied, but this limitation can also be seen as a strength, as it allows for a specific and targeted evaluation of the HRI. By using HEUROBOX in conjunction with other evaluation methods, evaluators can gain a comprehensive understanding of the strengths and weaknesses of the robot in question. Moreover, by using HEUROBOX to evaluate different robots or different versions of the same robot, evaluators can compare the results across different scenarios and identify patterns or trends over time, providing valuable insights into the progress of robot development.

## 7 Conclusion

The increasing number of robots being incorporated into industry highlights the necessity to have natural, fluid, and satisfying interaction between humans and robots. This paper presents a contribution to a new evaluation list through a set of heuristics to assess HRI. It is a tool that facilitates the evaluation of HRI and allows the identification of aspects for improvement in the system in terms of UX. However, in order to obtain a complete and real evaluation of the UX, it is appropriate to complement it with other UX evaluation techniques, such as usability tests. HEUROBOX consists of four principal categories: Safety, Functionality, Ergonomics, and Interfaces.

• Safety is a critical aspect of HRI, and it encompasses a range of design considerations aimed at preventing harm to both humans and robots. These include safety measures such as emergency stops, safety barriers, and collision detection systems. Additionally, the design of the robot should take into account the physical and cognitive limitations of the users and be able to adapt to unexpected situations.
• Ergonomics is concerned with the interaction between the user and the robot, taking into account factors such as posture, movement, and comfort. The design of the robot should ensure that users can operate it without experiencing undue physical strain or discomfort.
• Functionality is a key aspect of the robot’s design, as it determines the robot’s ability to perform the intended tasks efficiently and effectively. The functionality of the robot should be tailored to the specific context of use and should be able to handle any unexpected situations that may arise. The robot should also be able to perform tasks autonomously, with minimal human intervention, if necessary.
• Interfaces are a crucial category in HRI design, as they define the communication channels between the user and the robot. Interfaces encompass a range of design considerations, including visual, voice, gesture, and haptic interfaces. Visual interfaces should be designed to provide the necessary information in a clear and structured way, with simple graphics and icons. Voice interfaces should be designed to be natural and human-like, with structured dialog between the user and the system. Gesture interfaces should be intuitive and natural for the user to use, while haptic interfaces should provide sufficient tactile feedback to the user.

While these four categories are distinct, they are closely related, and each has an impact on the other. For example, safety is closely related to ergonomics, as a robot that is ergonomically designed is less likely to cause harm to the user. Additionally, functionality is closely related to interfaces, as the interfaces must be designed to enable the robot to perform its intended tasks efficiently and effectively. Finally, the UX and technology acceptance will be impacted by all four categories, as the robot’s safety, ergonomics, functionality, and interfaces will all influence how the interaction is perceived.

Depending on the complexity of the robotic system itself, the level of heuristics to be met may be different to generate a satisfactory experience. That is, a simpler system may meet only the Basic level of heuristics and generate a satisfactory experience, and a more complex system may meet also the basic Level of heuristics but still generates a frustrating experience for the user. That is why we also propose the Advanced Level, so it is the expert who must choose which is the most adequate for the robot to be studied. Nevertheless, it is proposed to make a holistic assessment when evaluating the UX in HRI, so that it considers the characteristics of the user, context, and system in an objective and subjective way, before, during, and after the interaction. Furthermore, it would be necessary to validate and correlate the level of fulfillment of the heuristics with the level of satisfaction and acceptance perceived by the users during the interaction.

As a future line, it would be interesting to establish a classification/standard based on the complexity, characteristics, functionalities, and objectives of the robot, in order to determine the level of heuristics it must meet. By creating such a system, organizations and designers could ensure that robots are designed and evaluated in a consistent and comprehensive manner, allowing for more effective and efficient interaction between humans and robots. This would also provide a clearer understanding of the capabilities and limitations of different types of robots, allowing for more informed decisions regarding their use and deployment in various industries and contexts. Ultimately, such a classification/norm would contribute to the continued development and improvement of HEUROBOX, as it would serve as a framework for evaluating and designing robots that are tailored to meet the needs and expectations of their human collaborators.

In order to comprehensively evaluate and design multimodal interfaces, it is imperative to carefully consider the distinctive requirements and characteristics associated with each modality within the specific context of the robot under evaluation. The evaluation process should primarily focus on assessing the robot’s ability to enable seamless communication and interaction across different interfaces. In this regard, HEUROBOX should enhance the multimodality section of the list by placing particular emphasis on the extent to which the combination of different interfaces effectively complements one another and contributes to substantial improvements in overall interaction and UX.

Also, the ethical implications of robots are becoming increasingly important. While robots can bring many benefits, they can also pose ethical challenges. In this regard, a future line of research could involve the development of an “ethical” category that would allow for the evaluation of whether the characteristics of the robot and context are ethical.

To finish with, our study acknowledges certain limitations that should be taken into account. Firstly, the selection of databases, although designed strategically to encompass a broad range of relevant publications, may have inadvertently excluded important articles from contributing to the research outcomes. Additionally, the selection and clustering of heuristics were subject to the researchers’ interpretation of the literature, potentially introducing bias despite efforts to mitigate it. Despite these limitations, our study provides valuable insights into the topic and serves as a foundation for future research in the field of multimodal interactions and interface design.

## Data Availability

The datasets presented in this study can be found in online repositories. The names of the repository/repositories and accession number(s) can be found below: https://data.mendeley.com/datasets/t2nwpwwsmg/1.
