# Supplementary material for: Development of a new set of Heuristics for the evaluation of Human-Robot Interaction in industrial settings: Heuristics Robots Experience (HEUROBOX)
Source: Front Robot AI. 2023 Aug 31;10:1227082. doi: 10.3389/frobt.2023.1227082 (PMC10501719; doi:10.3389/frobt.2023.1227082)
Supplement: Supplementary file 1 [file DataSheet1.DOCX]

Supplementary Material

The complete set of heuristics

# Safety

| Subcategory | Level | Code | Heuristic | Ref |
| --- | --- | --- | --- | --- |
| General | Basic evaluation | HSGB-1 | The system minimizes all the general hazards related to human-assembly parts interaction. | (Gualtieri et al., 2020a) |
|  |  | HSGB-2 | The system minimizes specific mechanical hazards related to the entrapment of human body parts. | (Gualtieri et al., 2020a, 2022) |
|  |  | HSGB-3 | The system minimizes specific mechanical hazards related to human–assembly parts interaction. | (Gualtieri et al., 2020a, 2022) |
|  |  | HSGB-4 | The system minimizes specific mechanical hazards related to robot system parts falling. | (Gualtieri et al., 2020a, 2022) |
|  |  | HSGB-5 | The system does not have a physical form that can induce injury. | (Tsui et al., 2010; Weiss et al., 2010) |
|  |  | HSGB-6 | The system does not have behaviors that can induce injury. | (Tsui et al., 2010; Weiss et al., 2010) |
|  |  | HSGB-7 | The system provides a fail-safe mechanism. | (Tsui et al., 2010) |
| Motion planning | Advanced evaluation | HSMA-1 | The system allows to set trajectories in such a way human body parts will not be easily hit by the robot systems. | (Gualtieri et al., 2020b) |
|  |  | HSMA-2 | The system allows setting trajectories in such a way human body parts will not be easily trapped between the robot systems and the elements of the workstation. | (Gualtieri et al., 2020b) |
|  |  | HSMA-3 | The system allows to limit velocities of moving parts. | (Gualtieri et al., 2020b) |
|  |  | HSMA-4 | The system allows limiting momentum, mechanical power, or energy as a function of masses and velocities. | (Gualtieri et al., 2020b) |
|  |  | HSMA-5 | The system has safe virtual plane systems or space-limiting functions which limit the robot to work in a defined volume. | (Gualtieri et al., 2020b) |
|  |  | HSMA-6 | The system has speed limits for quasi-static contact. | (Gualtieri et al., 2020b) |
|  |  | HSMA-7 | The forces and torques of the system are limited via SW. | (Gualtieri et al., 2020b) |
|  |  | HSMA-8 | The system uses safety-rated soft axis. | (Gualtieri et al., 2020b) |
|  |  | HSMA-9 | The systems speeds are limited for transient contact. | (Gualtieri et al., 2020b) |
|  |  | HSMA-10 | The robot must identify landmarks in the route description. | (Bauer et al., 2009) |
| Robot systems | Advanced evaluation | HSRA-1 | The system has round edges and corners. | (Gualtieri et al., 2020b) |
|  |  | HSRA-2 | The system has smooth surfaces. | (Gualtieri et al., 2020b) |
|  |  | HSRA-3 | The system allows to manage of energy absorption, enlarges energy transfer time, and reduces impact forces (provide padding, cushioning or deformable components). | (Gualtieri et al., 2020b) |
|  |  | HSRA-4 | The system allows to limit momentum, mechanical power, or energy as a function of masses and velocities. | (Gualtieri et al., 2020b) |
|  |  | HSRA-5 | The system uses sensing to anticipate or detect contact. | (Gualtieri et al., 2020b) |
|  |  | HSRA-6 | The end-effector is designed to provide protection from hazards associated with the workpiece. | (Gualtieri et al., 2020b) |
|  |  | HSRA-7 | The system avoids entrapment due to the moving cables of the robot systems. | (Gualtieri et al., 2020b) |
|  |  | HSRA-8 | The system avoids entrapment due to exposed parts of the robot systems. | (Gualtieri et al., 2020b) |
|  |  | HSRA-9 | The objects and obstacles into the system are highlighted. | (Gualtieri et al., 2020b) |
| Organizational measures | Advanced evaluation | HSOA-1 | Robot system motion is signaled/highlighted. | (Gualtieri et al., 2020b) |
|  |  | HSOA-2 | The transition between collaborative operations and other kind of operations is signaled. | (Gualtieri et al., 2020b) |
|  |  | HSOA-3 | The performance of the robotic system enables it to be monitored. | (Gualtieri et al., 2020b) |
|  |  | HSOA-4 | The system allows the establishment of access routes (e.g., the paths taken by operators moving materials to the collaborative workspace). | (Gualtieri et al., 2020b) |

# Ergonomics

| Subcategory | Level | Code | Heuristic | Ref |
| --- | --- | --- | --- | --- |
| Physical ergonomics | Basic evaluation | HEPB-1 | The system physics is designed with ergonomics and accessibility in mind. | (Drury et al., 2004) |
|  |  | HEPB-2 | The system minimizes the biomechanical overload of upper limbs related to repetitive tasks. | (Gualtieri et al., 2020a) |
|  |  | HEPB-3 | The system minimizes the biomechanical overload of whole body related to manual lifting/lowering of objects. | (Gualtieri et al., 2020a) |
|  |  | HEPB-4 | The system minimizes the bio-mechanical overload of head/neck/trunk/upper or lower limbs related to static or awkward working postures. | (Gualtieri et al., 2020a) |
|  | Advanced evaluation | HEPA-1 | The system avoids tasks which require the use of upper limbs for long time. | (Gualtieri et al., 2020a) |
|  |  | HEPA-2 | The system avoids tasks which require the elbows position above the shoulder level for quite all the time | (Gualtieri et al., 2020a) |
|  |  | HEPA-3 | The system avoids tasks which require the use of moderate and continuous force | (Gualtieri et al., 2020a) |
|  |  | HEPA-4 | The system avoids tasks which require force peaks | (Gualtieri et al., 2020a) |
|  |  | HEPA-5 | The system avoids tasks which require the need of grasping using the fingers tips (all kinds) for quite all the time | (Gualtieri et al., 2020a) |
|  |  | HEPA-6 | The system avoids tasks which require high frequency and similar movements of upper limbs | (Gualtieri et al., 2020a) |
|  |  | HEPA-7 | The system avoids tasks which require to maintain the workstation elements far to the body | (Gualtieri et al., 2020a) |
|  |  | HEPA-8 | The system avoids tasks which require a vertical displacement outside the range between hips and shoulders | (Gualtieri et al., 2020a) |
|  |  | HEPA-9 | The system avoids tasks which require frequent body movements | (Gualtieri et al., 2020a) |
|  |  | HEPA-10 | The system reduces the weight and/or support heavy equipment, devices and, in general, every workstation elements manipulated by the operators | (Gualtieri et al., 2020a) |
|  |  | HEPA-11 | The system avoids tasks which require an asymmetric posture of booth neck and trunk | (Gualtieri et al., 2020a) |
|  |  | HEPA-12 | The system avoids tasks which require unsupported trunk backward inclination or harsh flexion | (Gualtieri et al., 2020a) |
|  |  | HEPA-13 | The system avoids tasks which require neck extension or hash flexion during the assembly | (Gualtieri et al., 2020a) |
|  |  | HEPA-14 | The system avoids tasks which require unsupported head backward inclination or harsh inclination | (Gualtieri et al., 2020a) |
|  |  | HEPA-15 | The system avoids tasks which require a convex spinal curvature (if sitting) | (Gualtieri et al., 2020a) |
|  |  | HEPA-16 | The system avoids tasks which require awkward upper arm postures | (Gualtieri et al., 2020a) |
|  |  | HEPA-17 | The system avoids tasks which require raised shoulder | (Gualtieri et al., 2020a) |
|  |  | HEPA-18 | The system avoids tasks which require unsupported upper arm elevation | (Gualtieri et al., 2020a) |
|  |  | HEPA-19 | The system avoids tasks which require extreme elbow flexion/extension AND extreme forearm rotation | (Gualtieri et al., 2020a) |
|  |  | HEPA-20 | The system avoids tasks which require extreme wrist deviation | (Gualtieri et al., 2020a) |
|  |  | HEPA-21 | The system avoids tasks which require extreme knee flexion | (Gualtieri et al., 2020a) |
|  |  | HEPA-22 | The system avoids tasks which require knee not flexed in standing postures | (Gualtieri et al., 2020a) |
|  |  | HEPA-23 | The system avoids tasks which require not-neutral ankle position | (Gualtieri et al., 2020a) |
|  |  | HEPA-24 | The system avoids tasks which require kneeling or crouching | (Gualtieri et al., 2020a) |
|  |  | HEPA-25 | The system avoids which require very high knee angle (if sitting) | (Gualtieri et al., 2020a) |
| Cognitive ergonomics | Basic evaluation | HECB-1 | The system makes components well identifiable and distinguishable. | (Gualtieri et al., 2020a) |
|  |  | HECB-2 | The system makes components easy to orient, locate and fasten. | (Gualtieri et al., 2020a) |
|  |  | HECB-3 | The system makes the work intuitive (supports the formation of a mental model, reduces the choice reaction time, facilitates the leaning transfer, promotes similarity) | (Gualtieri et al., 2020a) |
|  |  | HECB-4 | The system maximizes the user’s trust. | (Weiss et al., 2010) |
|  |  | HECB-5 | The UI is designed with ergonomics and accessibility in mind, ensuring the cobots UI is comfortable to work with for the necessary duration. | (Frijns and Schmidbauer, 2021) |
|  |  | HECB-6 | The system maximizes operator psychological wellbeing and satisfaction. | (Gualtieri et al., 2020b) |
|  |  | HECB-7 | The system avoids high speed motions. | (Gualtieri et al., 2020a) |
|  |  | HECB-8 | The robot is as low as possible. | (Gualtieri et al., 2020a) |
|  |  | HECB-9 | The system informs operators about the robot speed. | (Gualtieri et al., 2020a) |
|  | Advanced evaluation | HECA-1 | The system makes menu items that need attention visually salient, do not attract attention unnecessarily. | (Frijns and Schmidbauer, 2021) |
|  |  | HECA-2 | The system provides fused sensor information to avoid making the user fuse the data mentally. | (Drury et al., 2004) |
|  |  | HECA-3 | The system supports the user in understanding the connection between user actions and system response, for instance by providing feedback and using appropriate terminology. | (Frijns and Schmidbauer, 2021) |
|  |  | HECA-4 | The system ensures the robot performs in accordance with polite social etiquette. | (Weiss et al., 2010) |
|  |  | HECA-5 | The system provides fused sensor information to lower the cognitive load on user. | (Adamides et al., 2014) |
|  |  | HECA-6 | The system reduces the number of assembly sub-systems. | (Gualtieri et al., 2020a) |
|  |  | HECA-7 | The system allows an immersive operator experience: sense of "being there". | (Lewis et al., 2014) |
|  |  | HECA-8 | The system implements smooth trajectories (which can be assimilate to natural human-arm motions) | (Gualtieri et al., 2020a) |
|  |  | HECA-9 | The system implements swing trajectories (not continuously straight) | (Gualtieri et al., 2020a) |
|  |  | HECA-10 | The system involves operators into the definition of layout and work activities | (Gualtieri et al., 2020a) |
|  |  | HECA-11 | The system avoids misalignment in operator and robot use of production resources (avoids inefficiency) | (Gualtieri et al., 2020a) |

# Functionality

| Subcategory | Level | Code | Heuristic | Ref. |
| --- | --- | --- | --- | --- |
| System | Basic evaluation | HFSB-1 | The interaction with the robot is consistent and predictable. | (Tsui et al., 2010; Frijns and Schmidbauer, 2021; Qbilat et al., 2021) |
|  |  | HFSB-2 | The system supports the user in understanding the way it works. | (Frijns and Schmidbauer, 2021) |
|  |  | HFSB-3 | The system programs, trajectories and configurations are editable by the user. | (Frijns and Schmidbauer, 2021) |
|  |  | HFSB-4 | The system enables users to change the UI according to their needs. | (Frijns and Schmidbauer, 2021) |
|  |  | HFSB-5 | The system provides fail-safe mechanisms. | (Tsui et al., 2010) |
|  |  | HFSB-6 | The system allows manual control to move around (mobile robotics). | (Lewis et al., 2014) |
|  | Advanced evaluation | HFSA-1 | The user can decide the interaction level, settings and actions, i.e., to integrate human input or to make the program fully automatic (flexibility of interaction). | (Weiss et al., 2010; Young et al., 2011; Adamides et al., 2014; Frijns and Schmidbauer, 2021; Qbilat et al., 2021) |
|  |  | HFSA-2 | The system's software integration is reconfigured easily after hardware exchange, i.e. scalability of the architecture. | (Keebler et al., 2012; Adamides et al., 2014; Frijns and Schmidbauer, 2021) |
|  |  | HFSA-3 | The system manages autonomously user attention across different modalities. | (Frijns and Schmidbauer, 2021) |
|  |  | HFSA-4 | The system allows users to switch between simple and more complex ways of programming the cobot. | (Frijns and Schmidbauer, 2021) |
|  |  | HFSA-5 | The system manipulates relationship between robot and world | (Adamides et al., 2014) |
|  |  | HFSA-6 | The system provides assistance with autonomous modes. | (Adamides et al., 2014) |
|  |  | HFSA-7 | The interface switches modes automatically when necessary. | (Clarkson and Arkin, 2007; Weiss et al., 2010) |
|  |  | HFSA-8 | Sensor and actuator capabilities are adequate for the system’s expected tasks and environment. | (Clarkson and Arkin, 2007) |
|  |  | HFSA-9 | The system accommodates the ability to choose among access devices. | (Weiss et al., 2010) |
|  |  | HFSA-10 | The system provides multiple ways to access a function. | (Weiss et al., 2010) |
|  |  | HFSA-12 | The system is rigidly designed with the appropriate amount of interaction. | (Young et al., 2011; Lewis et al., 2014) |
|  |  | HFSA-13 | The system shows new functionalities as robotic systems gain new hardware and software components. | (Keebler et al., 2012; Adamides et al., 2014; Frijns and Schmidbauer, 2021) |
| Information | Basic evaluation | HFIB-1 | User can operate the robot using different channels for input and output. | (Qbilat et al., 2021) |
|  |  | HFIB-2 | User can check and correct interaction information before submitting. | (Qbilat et al., 2021) |
|  |  | HFIB-3 | The system informs the user regarding the cobot’s environment (spacial information) and configuration (indicators of robot health/state). | (Drury et al., 2004; Tsui et al., 2010; Adamides et al., 2014; Lewis et al., 2014; Frijns and Schmidbauer, 2021) |
|  |  | HFIB-4 | The system allows users to access information required for the task, i.e., gives mission feedback. | (Clarkson and Arkin, 2007; Adamides et al., 2014; Frijns and Schmidbauer, 2021) |
|  |  | HFIB-5 | The system communicates to the user which task is being executed. | (Frijns and Schmidbauer, 2021) |
|  |  | HFIB-6 | The UI uses simple language to reduce mental processing. | (Tsui et al., 2010) |
|  |  | HFIB-7 | The system avoids having the user to make mental translations. | (Tsui et al., 2010) |
|  |  | HFIB-8 | The system provides effective (precise and accurate) communication architecture. | (Keebler et al., 2012; Adamides et al., 2014) |
|  |  | HFIB-9 | The system provides with large video windows that assist in the success of the task. | (Adamides et al., 2014) |
|  |  | HFIB-10 | The system uses a single monitor for the interface. | (Adamides et al., 2014) |
|  |  | HFIB-11 | The system gives continuous understanding of the overall mission and the moment-by-moment at a reasonable time. | (Clarkson and Arkin, 2007; Adamides et al., 2014) |
|  |  | HFIB-12 | The system informs about progress towards completing the mission. | (Adamides et al., 2014; Frijns and Schmidbauer, 2021) |
|  |  | HFIB-13 | The system provides consistency of information, i.e., a match between system and real-world information. | (Tsui et al., 2010; Adamides et al., 2014) |
|  | Advanced evaluation | HFIA-1 | User can always query what the robot is doing or processing. | (Qbilat et al., 2021) |
|  |  | HFIA-2 | The system minimizes the use of multiple windows, i.e., supports multiple information in a single window, if possible (e.g., multi robot systems). | (Drury et al., 2004; Adamides et al., 2014) |
|  |  | HFIA-3 | The interface shows consistency between robot behavior and what the operator has been led to believe based on the interface (actions). | (Tsui et al., 2010; Adamides et al., 2014; Frijns and Schmidbauer, 2021) |
|  |  | HFIA-4 | When multiple robots are available, the system allows to use one to view another. | (Adamides et al., 2014) |
|  |  | HFIA-5 | The system implicitly switches interfaces modality and autonomy. | (Tsui et al., 2010; Adamides et al., 2014) |
|  |  | HFIA-6 | The system conveys the information of the video stream with respect to robot orientation | (Adamides et al., 2014) |
|  |  | HFIA-7 | The system uses the principle of recognition over recall, improving users’ situational awareness via attention management, i.e., helps directing the operator’s focus of attention. | (Clarkson and Arkin, 2007; Adamides et al., 2014; Frijns and Schmidbauer, 2021) |
|  |  | HFIA-8 | The system provides option awareness to enable decision makers to know what courses of action are available, what their likelihoods of success are, and what their relative costs are. | (Tsui et al., 2010) |
|  |  | HFIA-9 | The system provides users with sufficient historical information to understand trends and make predictions. | (Tsui et al., 2010) |
|  |  | HFIA-10 | The system exploits existing systems' long-term and working memory as user support. | (Tsui et al., 2010) |
|  |  | HFIA-11 | The system provides knowledge in the interface so that people do not have to remember it. | (Tsui et al., 2010) |
|  |  | HFIA-12 | The system allows retention of user’s preferences. | (Tsui et al., 2010) |
|  |  | HFIA-13 | The system provides feedback and interaction according to user's technical abilities (skills) | (Tsui et al., 2010) |
|  |  | HFIA-14 | The system shows awareness for the robot's autonomy-influenced behaviors. | (Lewis et al., 2014) |
|  |  | HFIA-15 | The system provides templates, instructions or other clues that indicate how the cobot can be interacted with. | (Gualtieri et al., 2020a; Frijns and Schmidbauer, 2021) |
| Task | Basic evaluation | HFTB-1 | Time does not interfere on users’ ability to finish any interactive task with the robot. | (Qbilat et al., 2021) |
|  |  | HFTB-2 | The system helps minimising process length. | (Weiss et al., 2010) |
|  |  | HFTB-3 | The sequence of tasks is efficient. | Self-generated |
|  |  | HFTB-4 | The object recognition activities are simplified. | (Gualtieri et al., 2020a) |
|  |  | HFTB-5 | The feeding activities are simplified. | (Gualtieri et al., 2020a) |
|  |  | HFTB-6 | Handling activities are simplified. | (Gualtieri et al., 2020a) |
|  |  | HFTB-7 | Assembly activities are simplified. | (Gualtieri et al., 2020a) |
|  | Advanced evaluation | HFTA-1 | The system avoids unnecessary work on the user’s side by minimizing the number of steps required to achieve goals (e.g., recognition, feeding, handling or assembly), providing appropriate shortcuts. | (Weiss et al., 2010; Gualtieri et al., 2020a; Frijns and Schmidbauer, 2021) |
|  |  | HFTA-2 | The system enables reuse of their previous work or the work of others. | (Frijns and Schmidbauer, 2021) |
|  |  | HFTA-3 | The system architecture allows adaptation of the system to different types of tasks and application scenarios. | (Frijns and Schmidbauer, 2021) |
|  |  | HFTA-4 | The system exploits previous world knowledge if reasonable during task execution. | (Weiss et al., 2010) |
|  |  | HFTA-5 | The system integrates robots early in team formation so that roles can be discovered, and trust established quickly by human members. | (Keebler et al., 2012) |
| Error handling | Basic evaluation | HFEB-1 | The system uses visual mechanisms to show errors. | Self-generated |
|  |  | HFEB-2 | The system logs or stores captured errors. | (Weiss et al., 2010) |
|  |  | HFEB-3 | The interface helps to recognize and diagnose user or robot errors, using appropriate descriptions. | (Weiss et al., 2010; Adamides et al., 2014) |
|  |  | HFEB-4 | The system helps the user to recover from an error situation. | Self-generated |
|  |  | HFEB-5 | The system anticipates errors | (Wibowo et al., 2017) |
|  |  | HFEB-6 | Error messages or failures are not displayed using coding, machine, or programming languages | (Wibowo et al., 2017) |
|  |  | HFEB-7 | Error messages are displayed using human languages that are easy to understand, understood by the user | (Wibowo et al., 2017) |
|  | Advanced evaluation | HFEA-1 | For any blinking component on robot’s interface (lights, display contents, etc.) the blinking stops after a certain period, or can be switched off by user. | (Qbilat et al., 2021) |
|  |  | HFEA-2 | The interface helps to recover from errors made by the operator or the robot with explanations and steps. | (Adamides et al., 2014; Frijns and Schmidbauer, 2021) |
|  |  | HFEA-3 | The system provides a forgiving interface, allowing for reversible actions on the part of the operator or the robot as much as possible. | (Adamides et al., 2014) |
|  |  | HFEA-4 | The system has the ability to self-inspect the robot’s body for damages or entangled obstacles. | (Adamides et al., 2014) |
|  |  | HFEA-5 | The system presents enough information about the task environment so that the user can determine if some aspect of the world has contributed to the error. | (Clarkson and Arkin, 2007) |
|  |  | HFEA-6 | The system considers multiple sources of errors (communication errors, input errors, mode errors…). | (Weiss et al., 2010; Keebler et al., 2012) |
|  |  | HFEA-7 | Before performing the action, save option, edit option, delete option, search option or other option user will be reminded last action through message box. | (Wibowo et al., 2017) |
| Assistance | Basic evaluation | HFAB-1 | User can ask for robot help or support at any given time. | (Young et al., 2011; Qbilat et al., 2021) |
|  |  | HFAB-2 | The system provides clear information and feedback | (Wibowo et al., 2017) |
|  |  | HFAB-3 | The system uses standard rules for decision support | (Wibowo et al., 2017) |
|  | Advanced evaluation | HFAA-1 | The system provides clear contextual help and documentation of available functionalities and possible errors (context-sensitive help). | (Weiss et al., 2010; Frijns and Schmidbauer, 2021; Qbilat et al., 2021) |
|  |  | HFAA-2 | The system supports trial-and-error behaviors. | (Frijns and Schmidbauer, 2021) |
|  |  | HFAA-3 | The system provides the operator assistance in determining the most appropriate level of robotic autonomy (modality) at any given time (automation problem). | (Drury et al., 2004; Adamides et al., 2014; Frijns and Schmidbauer, 2021) |

# Interfaces

| Level | Subcategory | Code | Heuristic | Ref. |
| --- | --- | --- | --- | --- |
| Basic evaluation | General | HIG-1 | The UI supports the user in maintaining appropriate awareness of the system’s state. | (Frijns and Schmidbauer, 2021) |
|  |  | HIG-2 | The UI makes it easy for the user to follow task execution by indicating previous, current and next steps. | (Frijns and Schmidbauer, 2021) |
|  |  | HIG-3 | The UI limits the number of options that are presented. | (Frijns and Schmidbauer, 2021) |
|  |  | HIG-4 | The UI attracts attention with the proper mechanisms. | (Frijns and Schmidbauer, 2021) |
|  |  | HIG-5 | The system and the UI behave in a consistent way. | (Frijns and Schmidbauer, 2021) |
|  |  | HIG-6 | The UI is easy and intuitive to use. | (Adamides et al., 2014; Frijns and Schmidbauer, 2021) |
|  |  | HIG-7 | The design is intended for user's specificity, not the developer. | (Adamides et al., 2014) |
|  |  | HIG-8 | The UI grounds the information displayed with the reality. | (Adamides et al., 2014) |
|  |  | HIG-9 | The UI allows flexible, effective and efficient interactions. | (Adamides et al., 2014) |
|  |  | HIG-10 | The UI makes it obvious what actions are available at any given moment. | (Adamides et al., 2014) |
|  |  | HIG-11 | The UI follows real-world conventions. | (Adamides et al., 2014) |
|  |  | HIG-12 | System malfunctions are expressed in plain language (no codes). | (Clarkson and Arkin, 2007) |
|  |  | HIG-13 | The UI presents content appropriately. | (Weiss et al., 2010) |
|  |  | HIG-14 | The system avoids the overload of the user’s short-term memory, i.e., limiting the number of significant items (e.g., digits, letters, gestures) that must be remembered | (Coronado et al., 2017; Fulfagar et al., 2021) |
|  |  | HIG-15 | The UI is configured in different languages. | (Clarkson and Arkin, 2007) |
|  |  | HIG-16 | The UI enables easy recovery from errors and offer guidance to the user on how they can correct it. | (Fulfagar et al., 2021) |
|  |  | HIG-17 | The UI provides ability to control and interrupt: The system should allow the user to interrupt if routed to a path they do not wish to follow. | (Fulfagar et al., 2021) |
|  |  | HIG-18 | The system allows to use assistive technology to interact with the robot, such as screen reader, braille keyboards, etc. | (Qbilat et al., 2021) |
|  |  | HIG-19 | The UI indicates which actions are currently possible and which ones are not (affordances). | (Frijns and Schmidbauer, 2021) |
|  |  | HIG-20 | The UI tolerates minor user errors, prevent critical system errors, support undo and redo. | (Frijns and Schmidbauer, 2021) |
|  |  | HIG-21 | The UI gives users clear explanations of functionality and errors. | (Frijns and Schmidbauer, 2021) |
|  |  | HIG-22 | The UI supports easy editing of robot programs. | (Frijns and Schmidbauer, 2021) |
|  |  | HIG-23 | The UI ensures the way information is presented via different modalities consistently. | (Frijns and Schmidbauer, 2021) |
|  |  | HIG-24 | The UI and system blend together so the UI is an extension of the user, the system and by proxy, the world. | (Clarkson and Arkin, 2007) |
|  |  | HIG-25 | The physical embodiment of the system is pleasing in its intended setting. The UI do not contain information that is irrelevant or rarely needed. | (Clarkson and Arkin, 2007) |
|  |  | HIG-26 | User can easily perceive and access robot’s interfaces (hardware and software) components. | (Qbilat et al., 2021) |
|  |  | HIG-27 | If the system will be used over a lengthy period of time, the UI supports the evolution of system capabilities, such as sensor and actuator capacity, behavior changes and physical alteration. | (Clarkson and Arkin, 2007) |
|  |  | HIG-28 | Human and robot have a system of check-backs to ensure closed-loop communication (confirmation of reception). | (Keebler et al., 2012) |
| Advanced | Visual Interfaces | HIAV-1 | The UI shows information in a way that is easy for the user to follow task progression | (Frijns and Schmidbauer, 2021) |
|  |  | HIAV-2 | The UI avoids a complex design and makes use of simple graphics and icons. | (Frijns and Schmidbauer, 2021) |
|  |  | HIAV-3 | The UI minimizes the use of multiple windows. | (Adamides et al., 2014) |
|  |  | HIAV-4 | The UI presents the necessary information according to task, useful and relevant. | (Adamides et al., 2014) |
|  |  | HIAV-5 | The language of the interaction between the user and the system is in terms of words, phrases and concepts familiar to the user | (Clarkson and Arkin, 2007) |
|  |  | HIAV-6 | The design of the UI's items are usable, accessible, and aesthetic. | (Frijns and Schmidbauer, 2021) |
|  |  | HIAV-7 | The information is presented in a clear and structured way, and use color, contrast and salience appropriately. | (Frijns and Schmidbauer, 2021) |
|  |  | HIAV-8 | Fused information from different sensors are provided correctly. | (Drury et al., 2004) |
|  |  | HIAV-9 | The UI has an aesthetic and minimalist design (simple use). | (Weiss et al., 2010; Adamides et al., 2014; Frijns and Schmidbauer, 2021) |
|  |  | HIAV-10 | The UI displays the robot’s body. | (Adamides et al., 2014) |
|  |  | HIAV-11 | The UI presents automatic presentation of contextually appropriate information. | (Adamides et al., 2014) |
|  |  | HIAV-12 | The UI presents clear information in appropriate form. | (Adamides et al., 2014) |
|  |  | HIAV-13 | The UI prioritizes placement of information. | (Adamides et al., 2014) |
|  |  | HIAV-14 | The UI allows the user to manipulate the information displayed and to store information. | (Adamides et al., 2014) |
|  |  | HIAV-15 | The UI presents sensor information that is easily understood and in a useful form. | (Clarkson and Arkin, 2007) |
|  |  | HIAV-16 | The system displays programming functions at different levels of detail. | (Frijns and Schmidbauer, 2021) |
|  |  | HIAV-17 | Windows occlusion hinders operation. | (Adamides et al., 2014) |
|  |  | HIAV-18 | The UI presents information from multiple sensors presented in an integrated fashion. | (Adamides et al., 2014) |
|  |  | HIAV-19 | The UI has an easy transition to more in-depth information. | (Adamides et al., 2014) |
|  |  | HIAV-20 | The UI uses efficient interaction language, using natural human cues. | (Clarkson and Arkin, 2007; Adamides et al., 2014) |
|  |  | HIAV-21 | The GUI items are designed with usability, accessibility, and aesthetics in mind. The information is presented in a clear and structured way, and use color, contrast, and salience appropriately | (Frijns and Schmidbauer, 2021) |
|  |  | HIAV-22 | The UI color is not the only way to indicate hardware controls, keys and labels of the robot. This also applies to software widgets (buttons, labels, etc.) or for information displayed on the robot. | (Frijns and Schmidbauer, 2021) |
|  | Voice | HIAVo-1 | The UI allows the fewer number of steps that user-system dialog requires. | (Maguire, 2019) |
|  |  | HIAVo-2 | The UI ensures adequate system feedback. The system should always keep the user informed about what is going on through appropriate feedback within a reasonable time, providing, if necessary, confirmation of actions | (Maguire, 2019) |
|  |  | HIAVo-3 | The UI ensures high accuracy to minimize input errors. Recognition is important since errors degrade usability and lead to user frustration. | (Maguire, 2019) |
|  |  | HIAVo-4 | The UI is consistent and standard: Users should be able to maintain their focus on one interface or the link to a second interface (e.g., screen display) should be clear and consistent in operation. | (Maguire, 2019) |
|  |  | HIAVo-5 | The VUI should speak in a natural way and adopt human-to-human speech conventions. | (Maguire, 2019) |
|  |  | HIAVo-6 | The UI uses efficient interaction language, using natural human cues. | (Clarkson and Arkin, 2007; Adamides et al., 2014) |
|  |  | HIAVo-7 | The language of the interaction between the user and the system is in terms of words, phrases, and concepts familiar to the user | (Clarkson and Arkin, 2007) |
|  |  | HIAVo-8 | The dialog must be structured according to the four phases Introduction, Giving Directions, Confirmation, and Conclusion | (Bauer et al., 2009) |
|  |  | HIAVo-9 | The robot must interpret ’here’ and ’there’ depending on when they occur in the route description. | (Bauer et al., 2009) |
|  |  | HIAVo-10 | The robot must identify movement verbs. | (Bauer et al., 2009) |
|  |  | HIAVo-11 | The delimitation problem must be solved by modeling the distance ranges of various expressions such as ’here’, ’near’, and ’far’ depending on the environment. | (Bauer et al., 2009) |
|  | Gesture | HIAG-1 | The number of options involved in the interaction is as minimum as possible, to reduce the user’s response times and errors. | (Coronado et al., 2017) |
|  |  | HIAG-2 | The UI provides explicit hints to possible actions, guidelines on how to perform them, and direct feedback on the user’s performance, to enforce learnability | (Coronado et al., 2017) |
|  |  | HIAG-3 | The robot must solve the coordination problem, by asking the human to specify the first direction with a pointing gesture. | (Bauer et al., 2009) |
|  |  | HIAG-4 | The robot must interpret the basic directions ’left’, ’right’, ’straight’, and ’back’. | (Bauer et al., 2009) |
|  | Haptic | HIAH-1 | The texture of the tactile object is perceptible | (Andonovski et al., 2010) |
|  |  | HIAH-2 | The tactile object does not need too much force | (Andonovski et al., 2010) |
|  |  | HIAH-3 | The tactile objects’ level of vibration is significant. | (Andonovski et al., 2010) |
|  |  | HIAH-4 | The duration of a haptic/tactile object is sufficient for the user to perceive changes | (Andonovski et al., 2010) |
|  |  | HIAH-5 | The acceleration and the orientation speed of a tactile/haptic object are perceived and visualized. | (Andonovski et al., 2010) |
|  |  | HIAH-6 | Spatial attributes such as shape, location, and size exist for visual/graphic forms of interaction. | (Andonovski et al., 2010) |

# References

Adamides, G., Christou, G., Katsanos, C., Xenos, M., and Hadzilacos, T. (2014). Usability guidelines for the design of robot teleoperation: A taxonomy. *IEEE Trans Hum Mach Syst* 45, 256–262.

Andonovski, B., Ponsa, P., and Casals, A. (2010). Towards the development of a haptics guideline in human-robot systems. in *3rd International Conference on Human System Interaction* (IEEE), 380–387.

Bauer, A., Gonsior, B., Wollherr, D., and Buss, M. (2009). Heuristic rules for human-robot interaction based on principles from linguistics-asking for directions. in *AISB Convention-Symposium on New Frontiers in Human-Robot Interaction*, 24–30.

Clarkson, E., and Arkin, R. C. (2007). Applying Heuristic Evaluation to Human-Robot Interaction Systems. in *Flairs Conference*, 44–49.

Coronado, E., Villalobos, J., Bruno, B., and Mastrogiovanni, F. (2017). Gesture-based robot control: Design challenges and evaluation with humans. in *2017 IEEE international conference on robotics and automation (ICRA)* (IEEE), 2761–2767.

Drury, J. L., Hestand, D., Yanco, H. A., and Scholtz, J. (2004). Design guidelines for improved human-robot interaction. in *CHI’04 extended abstracts on Human factors in computing systems*, 1540.

Frijns, H. A., and Schmidbauer, C. (2021). Design Guidelines for Collaborative Industrial Robot User Interfaces. in *IFIP Conference on Human-Computer Interaction* (Springer), 407–427.

Fulfagar, L., Gupta, A., Mathur, A., and Shrivastava, A. (2021). Development and Evaluation of Usability Heuristics for Voice User Interfaces. in *International Conference on Research into Design. Smart Innovation, Systems and Technologies* (Springer, Singapore), 375–385. doi: 10.1007/978-981-16-0041-8_32.

Gualtieri, L., Monizza, G. P., Rauch, E., Vidoni, R., and Matt, D. T. (2020a). From Design for Assembly to Design for Collaborative Assembly - Product Design Principles for Enhancing Safety, Ergonomics and Efficiency in Human-Robot Collaboration. *Procedia CIRP* 91, 546–552. doi: 10.1016/J.PROCIR.2020.02.212.

Gualtieri, L., Rauch, E., and Vidoni, R. (2022). Development and validation of guidelines for safety in human-robot collaborative assembly systems. *Comput Ind Eng* 163, 107801. doi: 10.1016/j.cie.2021.107801.

Gualtieri, L., Rauch, E., Vidoni, R., and Matt, D. T. (2020b). Safety, Ergonomics and Efficiency in Human-Robot Collaborative Assembly: Design Guidelines and Requirements. *Procedia CIRP* 91, 367–372. doi: 10.1016/J.PROCIR.2020.02.188.

Keebler, J. R., Jentsch, F., Fincannon, T., and Hudson, I. (2012). Applying team heuristics to future human-robot systems. in *Proceedings of the seventh annual ACM/IEEE international conference on Human-Robot Interaction*, 169–170.

Lewis, T., Drury, J. L., and Brandon, B. (2014). Evaluating Mobile Remote Presence (MRP) Robots. in *Proceedings of the 18th International Conference on Supporting Group Work*.

Maguire, M. (2019). Development of a heuristic evaluation tool for voice user interfaces. in *International conference on human-computer interaction* (Springer), 212–225.

Qbilat, M., Iglesias, A., and Belpaeme, T. (2021). A Proposal of Accessibility Guidelines for Human-Robot Interaction. *Electronics 2021, Vol. 10, Page 561* 10, 561. doi: 10.3390/ELECTRONICS10050561.

Tsui, K. M., Abu-Zahra, K., Casipe, R., M’Sadoques, J., and Drury, J. L. (2010). Developing heuristics for assistive robotics. in *2010 5th ACM/IEEE International Conference on Human-Robot Interaction (HRI)* (IEEE), 193–194.

Weiss, A., Wurhofer, D., Bernhaupt, R., Altmaninger, M., and Tscheligi, M. (2010). A methodological adaptation for heuristic evaluation of HRI. in *19th International Symposium in Robot and Human Interactive Communication* (IEEE), 1–6.

Wibowo, R. M., Erna, P. A., and Hidayah, I. (2017). Heuristic evaluation and user testing with ISO 9126 in evaluating of decision support system for recommendation of outstanding marketing officer. in *2017 International Conference on Sustainable Information Engineering and Technology (SIET)* (IEEE), 454–458.

Young, J. E., Sung, J., Voida, A., Sharlin, E., Igarashi, T., Christensen, H. I., et al. (2011). Evaluating human-robot interaction: Focusing on the holistic interaction experience. *Int J Soc Robot* 3, 53–67. doi: 10.1007/S12369-010-0081-8.
